# Inflammatory cytokines in type 2 diabetes mellitus as facilitators of hypercoagulation and abnormal clot formation

**DOI:** 10.1186/s12933-019-0870-9

**Published:** 2019-06-04

**Authors:** Shehan N. Randeria, Greig J. A. Thomson, Theo A. Nell, Timothy Roberts, Etheresia Pretorius

**Affiliations:** 1 Department of Physiological Sciences, Stellenbosch University, Private Bag X1, Matieland, 7602 South Africa; 2 Department of Biochemistry, Institute of Integrative Biology, Faculty of Health and Life Sciences, University of Liverpool, Crown St, Liverpool, L69 7ZB UK

**Keywords:** Type 2 diabetes mellitus, Vascular dysfunction, Biomarkers, Systemic inflammation

## Abstract

**Background:**

The global burden of type 2 diabetes mellitus (T2DM), together with the presence of cardiovascular risk in this population, is reaching pandemic levels. A prominent feature of T2DM is chronic and systemic inflammation, with the accompanying presence of circulating and dysregulated inflammatory biomarkers; which in turn is associated with abnormal clot formation.

**Methods:**

Here, we investigate the correlation between abnormal blood clotting, using thromboelastography (TEG), clot ultrastructure using scanning electron microscopy (SEM) and the presence of a dysregulated inflammatory cytokine profile, by examining various circulating biomarkers.

**Results:**

Our results show that many biomarkers, across TEG, cytokine and lipid groups, were greatly dysregulated in the T2DM sample. Furthermore, our T2DM sample’s coagulation profiles were significantly more hypercoagulable when compared to our heathy sample, and ultrastructural analysis confirmed a matted and denser clot structure in the T2DM sample.

**Conclusions:**

We suggest that dysregulated circulating molecules may in part be responsible for a hypercoagulable state and vascular dysfunction in the T2DM sample. We propose further that a personalized approach could be of great value when planning treatment and tracking the patient health status after embarking on a treatment regimes, and that looking to novel inflammatory and vascular biomarkers might be crucial.

**Electronic supplementary material:**

The online version of this article (10.1186/s12933-019-0870-9) contains supplementary material, which is available to authorized users.

## Background

The global burden of diabetes was estimated at ~ 420 million during 2014 and has doubled since 1980 where the prevalence was ~ 5% compared to ~ 9% for adults diagnosed with type-2 diabetes mellitus (T2DM) [1]. Premature mortality rates resulting from T2DM and thromboembolic cardiovascular disease, are estimated to be as high as 43% in both men and women before the age of 70 years [2–5]. A most prominent feature of this condition is chronic (low-grade) inflammation, with the accompanying presence of circulating and dysregulated inflammatory biomarkers. Such a dysregulated circulating inflammatory marker profile is associated both with a hypercoagulable state and hypofibrinolysis [6, 7]. Obesity, which is strongly associated with the prevalence and incidence of T2DM, has been shown to induce low grade (chronic) inflammation [8–10] and insulin resistance (IR) [11, 12]. Chronic multifactorial inflammation originating mostly from an imbalanced diet and lack of physical exercise, contribute towards the development of T2DM, and is well-known in known inflammatory disease [13]. Therefore, the complications as a result of low-grade inflammation and diet in T2DM, include mainly cardiovascular disease (CVD), mainly due to hyperglycaemia, and in turn might enhance dysregulated coagulation [2, 14]. Ultimately, an important determinant behind the current global T2DM epidemic is IR in overweight and obese individuals [15].

For T2DM patients disease profiling, clinicians typically measure C-reactive protein (CRP or highly sensitive (hs)-CRP), blood lipid profiles and glucose/Hb1Ac levels. Measuring CRP and lipid profiles have been shown to predict the development of T2DM in non-diabetics irrespective of adiposity and IR [16]. Traditionally, chronically elevated blood glucose concentrations are indicative of T2DM risk [15] and both a persistent dysregulated blood glucose level and abnormal lipid profile would typically confirm T2DM. Type 2 diabetic individuals also have a greater risk for cardiovascular complications [17]. Other measures such as blood pressure and BMI would typically also be taken into consideration when a treatment regime is planned. There is a plethora of evidence describing the correlation between CRP, monocyte chemoattractant protein-1 (MCP-1), tumor necrosis factor-α (TNF-α) and IL-1β in obese T2DM patients [18, 19].

Due to the pandemic nature of T2DM, and increased cardiovascular risk, there is a growing need for innovative strategies to change our approach in both the identification and successful treatment of such individuals. A much more focussed approach is therefore needed in patient care of T2DM, by regularly assessing systemic inflammation and cardiovascular risk in this population, and one such approach would be to turn to the use of circulating biomarkers to track disease progression, as well as medication effectiveness.

Inflammatory (circulating) biomarkers, when dysregulated, have been identified as key players in causing abnormal clot formation (see Additional file 1: Tables S1 and S2, for a summary of inflammatory biomarkers as well as their role in blood dysregulated blood clotting (both aberrant hypercoagulation and hypofibrinolysis) both in disease and experimental inquisition.

Mutie et al. [15] argue that the emergence of biomarker technologies has allowed more targeted therapeutic strategies (precision medicine) for diabetes prevention; however, currently, the use of such technologies are largely confined to pharmacotherapy. Although precision diabetes medicine is confined to pharmacotherapy, using biomarkers to personalise lifestyle recommendations, in T2DM treatment is becoming a real possibility [20]. Both personalised and precision medicine approaches in T2DM should therefore be an important and urgent consideration, as many individuals with T2DM have multiple (cardiovascular) comorbidities [21].

The current paper investigates hypercoagulation, using thromboelastography of platelet poor plasma (PPP), and PPP clot ultrastructure, using clots prepared with thrombin, and viewed with a scanning electron microscope in healthy and T2DM individuals. Furthermore, we study levels of 20 circulating cytokine biomarkers and how they might differ between healthy individuals, and individuals with T2DM. We also look at intercellular adhesion molecule-1 (ICAM-1), vascular adhesion molecule-1 (VCAM-1), and serum amyloid A (SAA). The increase in the concentrations of (soluble) sVCAM-1 and sICAM-1 reflects the degree of endothelial damage, and ultimately may impact on cardiovascular risk if it is chronically increased. Levels of SAA, which is a major acute phase protein in humans, are increased up to 1000-fold upon infection, trauma, cancer or other inflammatory events, and it plays a key role in regulating the inflammatory response [22]. Dysregulation of the circulating cytokines that we measure in this paper, are also closely linked to dysregulation of sICAM-1, sVCAM-1 and SAA. The pro-inflammatory cytokines we included in this study, have also been associated with platelet (hyper) activation, as well as erythrocyte dysfunction, while CRP dysregulation is directly linked to dysregulation of some of these molecules, and a strong indication of systemic inflammation. CRP is a part of the member of the pentraxin family which are pattern recognition proteins that play an integral role in the innate immune system [23]. When secreted from the liver in response to IL-6, CRP forms an annular pentameric protein shape which then circulates throughout the body in blood plasma [24]. Interleukin-6 (IL-6) and its downstream product, CRP, are therefore both well-known inflammatory molecules. Importantly, due to an increase in IL-6, circulaitng CRP levels can increase up to 1000 times during disease or inflammation [25]. We also included analysis of levels of HbA1c and lipid profiles, as these are markers that can confirm hyperglycemia and systemic inflammation.

The following paragraphs briefly discuss some of the main interactions between circulating cytokines and sICAM-1, sVCAM-1 and SAA. *ICAM*-*1* is referred to as Cluster of Differentiation 54(CD54), is a protein encoded by the ICAM1 gene which encodes for a glycoprotein located on the cell surface of endothelial cells, as well as cells of the immune system, such as leukocytes [26, 27]. These cells continuously present with ICAM-1 at low levels on their cell membranes, however, when stimulated via TNF-α [28, 29], IL-1β, lipopolysaccharide (LPS) [30] and the presence of reactive oxygen species (ROS) [31], ICAM-1 concentrations greatly increase. ICAM-1 levels have been found to be significantly elevated in T2DM [32], and elevated ICAM-1 may be as a result of elevated TNF-α and IL-1β found in T2DM. However, the increased ICAM-1 may influence T2DM pathogenesis via the binding of monocytes/leukocytes to activated vascular endothelium preceding macrophage and foam cell development [33], a crucial event potentially resulting in T2DM.

VCAM-1, otherwise known as cluster of differentiation 106 (CD106) is a 90-kDa glycoprotein encoded by the VCAM-1 gene [34], may also be of importance in inflammation. VCAM-1 expression similar to ICAM-1, is activated and expressed on the cell membrane of endothelial cells [35] in the presence of various pro-inflammatory cytokines such as TNF-α as well as the presence of ROS [36]. When stimulated by circulating inflammatory cytokines, various ligands bind to the externalised VCAM-1, causing leukocyte to endothelial cell binding which starts a cascade effect whereby ending in VCAM-1-dependent leukocyte trans-endothelial migration [37]. VCAM-1 is also significantly elevated in T2DM [38, 39] and may be one of the leading causes of the abnormal endothelial function and activation that occurs in T2DM [40, 41]. CRP levels are significantly elevated in T2DM and is a strong biomarker for T2DM disease progression and management [42]. SAA presence is also associated with the pathogenesis of chronic inflammatory diseases, such as T2DM, as well as atherosclerosis [43, 44].

Platelet (dys)function and both ICAM-1 and VCAM-1 are also closely linked. Platelet activation and endothelial damage play essential roles in atherosclerosis and thrombin-activated platelet-derived exosomes regulate endothelial cell expression of ICAM-1 [45]. Proteins that are essential for platelet adhesion include P-selectin, von Willebrand factor (vWF), and ICAM-1 [45]. During activation, platelets release thromboxane A2 (TXA2), which may also bind to receptors on platelet membranes. Also binding of locally produced TXA2 to thromboxane receptors on endothelial cells causes upregulation of ICAM-1 on the cell surface [46]. P-selectin mediates platelet rolling on vascular wall endothelial cells [47], furthermore von Willebrand factor (vWF) is required for platelet adhesion through the platelet receptor GPIbα, and ICAM-1 has been reported to mediate the platelet receptor GPIIb/IIIa-dependent bridging mechanism through fibrinogen [48, 49]. Another platelet receptor of interest if GPVI, which is a collagen receptor on platelets, and it is known that collagen is exposed from endothelial cells, in cardiovascular diseases, and conditions like T2DM. It has been found that there is a positive correlation between a dysregulation between surface expression of platelet GPVI and with the level of ICAM-1 in endothelial cells in disease [50]. Furthermore, platelets tether and roll on P-selectin presented on activated endothelium via P-selectin GP ligand‐1 (PSGL)-1 and GPlb, and arrest via GPIIb–IIIa and fibronectin on endothelial αvβ3 and ICAM-1 [46]. Platelets constitute the main source of soluble CD40 ligand (sCD40L), expressed on the platelet surface under basal conditions and additionally translocates to the plasma membrane upon activation. sCD40L induces endothelial upregulation of ICAM‐1, VCAM‐1, and E‐selectin, and thus supports leukocyte recruitment.

Serum amyloid A (SAA) is a generic term for a highly conserved family of acute-phase apolipoproteins synthesised by the liver [43, 51]. The SAA molecules can be divided into two groups, namely the acute phase SAAs that associate with HDL during inflammation; and constitutive SAA, including human SAA4 [52]. Human apo-SAA is a 104 amino acid polypeptide that circulates in plasma bound to high density lipoprotein-3 (HDL3) [53]. However, SAA has an important effect on HDL structure and function during inflammation, as the majority of SAA is an apolipoprotein of high-density lipoprotein HDL [54, 55]. During acute inflammation, SAA secreted from the liver displaces apolipoprotein A-I bound to HDL, with each HDL particle being able to bind and carry several copies of SAA [56], thus becoming the major apolipoprotein of circulating HDL3 [43]. Acute-phase SAA also modifies the biological effects of HDL-C in several conditions [57]. Depending on the extent of inflammation, it has been seen that SAA may increase up to 1000-fold, compared to those in the non-inflammatory state [43]. Consequently, SAA is a well-established (and potent) biomarker for infection and sepsis [58–60]. However, increased SAA is also an important plasma biomarker for predicting future cardiovascular events, and is associated with an increase in thrombotic risk [61, 62] as it is an active participant in the early atherogenic process [63].

CRP is also a known (hyper) activating ligand via platelet receptors and various platelet receptors form complexes on ligand binding, including GPVI with FcRγ (chain) after fibrin (ogen) [64–67], collagen, CRP and thrombin binding [68]. After CRP binds to the GPVI/FcRγ (chain) complex, a cascade follows, resulting in shedding by metalloproteases such as a-disintegrin-and-metalloproteinase 10 (ADAM10) [69]. SAA may also cause platelet (hyper) activation. We established that free SAA, added at low physiological levels (30 µg mL^−1^), representing a modest acute phase response, caused red blood cell agglutination, platelet activation and aggregation, but without platelet spreading [70]. It was suggested that the highly lipophilic SAA interacts within the platelet cell surface phospholipids (analogous to HDL) to produce these effects. The correlations between vascular injury, circulating dysregulated cytokines and hypercoagulation may therefore be of great importance and point to greater cardiovascular risk in T2DM, and might be used to track disease severity for a more individualized approach in T2DM care.

## Material and materials

### Ethics, consent and permissions

The study received ethical clearance from the Health Research Ethics Committee (HREC) of Stellenbosch University (South Africa) (approval number N17/01/008_RECIP_UP.298/2016: E Pretorius) and S18/02/036: G Thomson and E Pretorius. Prior to the commencement of the study, all volunteers were informed of procedures and data collection. Written informed consent was obtained from all participants followed by whole blood sampling in citrated tubes. All participants received a unique number that was used to ensure confidentiality throughout the study. All investigators were certified in Good Clinical Practice and ethical codes of conduct.

### Study design, setting and study population

A cross-sectional design was followed in collaboration with an endocrinologist and physician, who provided whole blood (WB) from T2DM private patients.

#### Healthy control sample

Whole blood from healthy controls were collected by a Health Professions Council of South Africa (HPCSA) registered Medical Biological Scientist and phlebotomist (MW: 0010782) at the Department of Physiological Sciences, Stellenbosch University. The total study population consisted of n = 87 volunteers (n = 46 healthy controls, and n = 41 T2DM patients), from which the 20-Plex cytokine analysis, thromboelastography, lipogram and V-Plex vascular injury analysis were conducted.

#### Type 2 diabetes mellitus sample

Whole-blood samples were also collected from individuals diagnosed with T2DM and cardiovascular disease at least 3 months prior to screening and without any clinical indication of infection (Society for Endocrinology, Metabolism and Diabetes of South Africa (SEMSDA) guidelines based on American Diabetes Association (ADA) criteria). Diabetic volunteers were recruited with the following inclusion criteria: (i) a confirmed diagnosis of T2DM with cardiovascular disease (ii) males and females older than 35 years. Similarly, healthy controls were included if they were (iii) non-smokers, (iv) not on any contraceptives, (v) not pregnant and/or lactating. Participants who were unable to provide written consent were excluded from the study. To limit and exclude confounding factors, both healthy and diabetic volunteers were only included if they are not diagnosed with tuberculosis, human immunodeficiency virus (HIV) or any malignancies. The inclusion criteria for healthy age-matched volunteers included: (i) no use of chronic medication (ii) no prior history of thrombotic disease or inflammatory conditions (iii) non-smokers, (iv) not on any chronic antiplatelet therapy/anticoagulant medication or any contraceptive/hormone replacement therapy (v) were not pregnant and/or lactating. Participants who were unable to provide written consent were also excluded from the study.

### Collection of whole blood (WB) and preparation of platelet poor plasma (PPP) samples from healthy controls and T2DM patients

Whole blood from heathy controls and T2DM patients were collected using sterile sampling techniques in citrate and ethylenediaminetetraacetic acid (EDTA) tubes, as well as serum separating tubes (SST) that were kept at room temperature (22 °C) for 30 min. Platelet poor plasma (PPP) were prepared from citrate tubes that were centrifuged at 3000×*g* for 10 min at room temperature (∼ 22 °C). The PPP was subsequently aliquotted into labelled 1.5 mL Eppendorf tubes, and stored at − 80 °C until laboratory analysis. EDTA whole blood and SST were analysed by the local PathCare laboratory (Stellenbosch) for glycosylated haemoglobin (HbA1c).

### Thromboelastography (TEG) of platelet poor plasma (PPP)

Clot kinetics/property studies was performed using Thromboelastography (TEG) (Thromboelastograph 5000 Hemostasis Analyzer System), on both control (n = 23) and T2DM (n = 25) PPP samples. After collection and PPP preparation, all samples were stored at − 80 °C until TEG analysis. At the time of TEG analysis, PPP samples were thawed from − 80 °C to room temperature. Once samples were completely thawed, 340 μL of PPP were placed in a disposable TEG cup to which 20 μL of 0.2 mol L^−1^ CaCl_2_ was added. CaCl_2_ is necessary to reverse the effect of the sodium citrate anticoagulant in the collection tube (i.e. recalcification of blood) and consequently initiate coagulation. The samples were then loaded in the TEG for analysis. The clot parameters that were assessed are presented in Table 1.

**Table 1:** Thromboelastography clot parameters for WB and PPP

Parameters	Explanation
R value: reaction time; measured in minutes	Time of latency from start of test to initial fibrin formation (amplitude of 2 mm); i.e. initiation time
K value: kinetics; measured in minutes	Time taken to achieve a certain level of clot strength (amplitude of 20 mm); i.e. amplification
Angle (Α/Alpha): slope between the traces represented by R and K; measured in degrees	The angle measures the speed at which fibrin build up and cross linking takes place, hence assesses the rate of clot formation; i.e. thrombin burst
Maximal amplitude (MA): measured in mm	Maximum strength/stiffness of clot. Reflects the ultimate strength of the fibrin clot, i.e. overall stability of the clot
Maximum rate of thrombus generation (MRTG): measured in Dyn cm^−2^ s^−1^	The maximum velocity of clot growth observed or maximum rate of thrombus generation using G, where G is the elastic modulus strength of the thrombus in dynes per cm^−2^
Time to maximum rate of thrombus generation (TMRTG): measured in minutes	The time interval observed before the maximum speed of the clot growth
Total thrombus generation (TTG): measured in Dyn cm^−2^	The clot strength: the amount of total resistance (to movement of the cup and pin) generated during clot formation. This is the total area under the velocity curve during clot growth, representing the amount of clot strength generated during clot growth
Coagulation index (CI)	Overall assessment of coagulability

### Scanning electron microscopy

In order to form the fibrin clots, 5 µL of purified thrombin (provided by the South African National Blood Service) was added into 10 µL of PPP from healthy controls (n = 10) and T2DM (n = 10) individuals on 10 mm round glass slides. Immediately after adding the thrombin, the glass slides were washed with 10× Gibco™ PBS (phosphate-buffered saline), pH 7.4 (ThermoFisher Scientific, 11594516) before fixing with 4% paraformaldehyde for a minimum of 30 min. Once fixed, samples were washed 3 × 3 min with PBS before a further 30-min fixation step in 1% osmium tetroxide (Sigma-Aldrich, 75632). A final 3 × 3 min PBS wash step was performed before samples were serially dehydrated in ethanol (30%, 50%, 70%, 90% and 100%) with a final 30-min dehydration step occurring using 99.9% hexamethyldisilazane (HMDS) ReagentPlus^®^ (Sigma-Aldrich, 379212). Samples were left in a fume hood to air-dry overnight before being mounted onto a microscope slide covered in double sided carbon tape. Samples were then carbon coated before being imaged on Zeiss MERLIN™ field emission scanning microscope located in the Central Analytical Facility (CAF) Electron Microbeam Unit, Stellenbosch University in order to determine fibrin(ogen) ultrastructure. All Micrographs were captured using the high resolution InLens capabilities at 1 kV.

### 20-Plex cytokine analysis

Stored PPP samples from control (n = 23) and T2DM (n = 25) volunteers were transferred from − 80 to − 20 °C 24 h prior to multiplex analysis. The samples were analysed in duplicate by means of Invitrogen’s Inflammation 20-Plex Human ProcartaPlex™ Panel (#EPX200-12185-901) and read on the Bio-Plex^®^ 200 system (Bio-Rad, 2016). The data is expressed in pg mL^−1^. See Table 2 for the list of pro-and anti-inflammatory cytokines that were analysed.

**Table 2:** List of pro- and anti-inflammatory biomarkers analyses with 20-plex multiplex kit

Anti-inflammatory biomarkers	
IFN-α	Interferon-alpha
IL-10	Interleukin-10
IL-13	Interleukin-13
IL-4	Interleukin-4
Pro-inflammatory biomarkers	
E-Selectin	E-Selectin
GM-CSF	Granulocyte-macrophage colony-stimulating factor
IFN-γ	Interferon-gamma
IL-1α	Interleukin-1 alpha
IL-1β	Interleukin-1 beta
IL-12p70	Interleukin-12p70
IL-17A	Interleukin-17A
IL-6	Interleukin-6
IL-8	Interleukin-8
IP-10	Interferon gamma-induced protein-10
MCP-1	Monocyte chemoattractant protein-1
MIP-1α	macrophage inflammatory protein-1 alpha
MIP-1β	macrophage inflammatory protein-1 beta
P-Selectin	P-Selectin
sICAM-1	Soluble intercellular adhesion molecule-1
TNF-α	Tumor necrosis factor-alpha

### V-Plex vascular injury analysis

This ELISA measures sICAM-1, sVCAM-1, SAA and CRP. Stored PPP samples from control (n = 36) and T2DM (n = 38) volunteers were transferred from − 80 to − 20 °C 24 h prior to multiplex analysis. The samples were analysed in duplicate by means of Meso Scale Discover Vascular Injury Panel 2 (human) Kits (catalogue number: K15198D-1) and read on the MSD Discovery Workbench 4 machine. The data is expressed in pg mL^−1^.

### Blood lipid analysis

Blood lipids of PPP samples from control (n = 23) and T2DM (n = 25) volunteers were analysed using the CardioChek Plus Professional Cholesterol/Glucose Analyzer (CardioChek Version 1.09, Polymer Technology Systems, United States of America). Total cholesterol (TC), LDL cholesterol, high-density lipoprotein (HDL) cholesterol, triglycerides (TG) and non-high-density lipoprotein (non-HDL) were measured, and TC/HDL ratio was calculated as a marker of cardiovascular disease (CVD) risk.

### Statistical analysis

All statistical analyses were performed using R version 3.5.3 and the built-in glm package. An age and gender adjusted logistic regression model was fit to both the main and expanded vascular injury datasets. Odds ratios (OR) and 95% confidence intervals, calculated using profiling, are reported. Mann–Whitney non-parametric tests were also performed, and P-values reported alongside logistic OR values. P-values of < 0.05 were accepted as statistically significant.

## Results

Demographic data including age, gender and medication used for typical comorbidities of T2DM patients were recorded by the medical professional on-hand on the day of blood sampling at the clinic. Similar demographic data for healthy controls were recorded prior to blood sampling (refer to Table 3 for sample demographics and data on medication usage) and TEG results of PPP samples are shown in Table 4.

**Table 3:** Sample demographics of study population, healthy and diabetic, and medication profile of diabetic volunteers

(a) Sample demographics of 20-Plex cytokine analysis, thromboelastography and lipogram analysis, healthy (n = 23) and diabetic (n = 25) volunteers			
	Healthy individuals (n = 23)	Diabetic individuals (n = 25)	P-values
Gender	Male (n = 14)	Male (n = 13)	
	Female (n = 9)	Female (n = 12)	
Age (years)	55.30 ± 3.59	59.75 ± 3.07	
HbA1c (%)	5.43 ± 0.10	9.05 ± 0.58	< 0.0001
Diabetic medication			
Hyperglycaemia medication	–	n = 17 (68.00%)	
Hypertension medication	–	n = 16 (64.00%)	
Hyperlipidaemia medication	–	n = 9 (36.00%)	
Anticoagulant medication	–	n = 12 (48.00%)	

(b) Sample demographics of V-Plex vascular injury analysis, healthy (n = 36) and diabetic (n = 38) volunteers			
	Healthy individuals (n = 36)	Diabetic individuals (n = 38)	P-values
Gender	Male (n = 16)	Male (n = 19)	
	Female (n = 20)	Female (n = 19)	
Age (years)	58.11 ± 10.98	64.58 ± 12.42	
HbA1c (%)	5.25 ± 0.09	8.96 ± 0.46	< 0.0001
Diabetic medication			
Hyperglycaemia medication	–	n = 30 (83.33%)	
Hypertension medication	–	n = 25 (69.44%)	
Hyperlipidaemia medication	–	n = 18 (50.00%)	
Anticoagulant medication	–	n = 19 (52.78%)	

Data expressed as mean ± SEM. No significant correlation was observed between age and HbA1c between the healthy and diabetic samples (Spearman-test) of both 20-Plex (and V-Plex analysis. Medications prescribed and used by diabetic volunteers were recorded, where *n* is the number of diabetic individuals on specific medication. Medication prescribed for hyperglycaemia included; Diamacron, Diaglucide Metformin, Diaphage, Pioglitazone, Galvus and insulin injections (Optisulin, Actraphane, Protaphane, Novomix, Apidra, Lantus, Actrapid, LEVEMIR^®^, Humulin). Medication prescribed for blood pressure regulation included; Zartan, Ridaq, Coversyl, Amlodipine, Pritor, LASIX^®^ Retard, Ciplavasc, Adco-Zetomax, Indapamide, Carvedilol, Carloc, Amloc Tareg, Adalat, Ciplazar, Spiractin, Irbesartan, Verahexal SR. Medication prescribed for cholesterol regulation included; Simvas, Adco Simvastatintatin, Atorvastatin, Vusor, Aspavor, Bezafibrate.; anticoagulant medication consisted of; Xarelto, Clexane, Aspirin, Heparin, Clopidogrel, Clexane, Ecotrin, novel oral anticoagulants

**Table 4:** Thromboelastography results of eight viscoelastic parameters assessing coagulation efficiency in naïve platelet poor plasma samples of healthy (n = 23) and T2DM (n = 25) volunteers

TEG clot parameters	Controls (n = 23)	T2DM (n = 25)	Adjusted OR (95% CI)	Mann–Whitney P-value
R-value (min)	9.6 [7.4 to 12.3]	7.5 [5.8 to 9.5]	*0.787 (0.616, 0.969)*	*0.032*
K-value (min)	3.75 [2.7 to 5.35]	2.15 [1.8 to 3.1]	0.765 (0.538, 1.03)	*0.011*
A angle (°)	61 [55.7 to 66.4]	68.1 [61.6 to 70.7]	1.06 (0.966, 1.17)	0.060
MA (mm)	23 [20.2 to 27.5]	30.3 [24.7 to 36.5]	*1.12 (1.03, 1.25)*	*0.011*
MRTG (Dyn cm^−2^ s^−1^)	3.25 [2.72 to 4.6]	5.45 [3.5 to 7.]	*1.59 (1.14, 2.39)*	*0.014*
TMRTG (min)	11.5 [8.5 to 14.7]	8.83 [7.5 to 12.2]	0.867 (0.714, 1.03)	0.100
TTG (Dyn cm^−2^)	149.4 [127 to 191]	218 [165 to 288]	1.01 (1, 1.02)	*0.011*
CI	− 4.55 [− 4.9 to − 4]	− 3.3 [− 4.1 to − 2.3]	*3.44 (1.55, 10.8)*	*0.006*

Summary statistics are given as median and IQR. Significant differences are shown in italics for both models

The results of the TEG analysis showed significant differences between the populations for six of the eight assessed parameters (Table 4, final column) and strong associations based on the adjusted logistic model in four of the parameters (4th column).

It is important to note that a subset of our T2DM samples were on anticoagulant medication, such as heparin and aspirin (see Table 3). Despite this, the diabetic individuals showed a significantly increased rate of clot formation (R-value), the initial rate of thrombus formation, as well as the K-value, which indicates the clot kinetics. Furthermore, in comparison to the healthy control group the T2DM individuals presented with a significantly elevated alpha angle (A angle), maximum amplitude (MA), maximal rate of thrombus generation (MRTG) as well total thrombus generation (TTG) values. This significant difference indicates that the clots formed faster in the diabetic sample with an overall hypercoagulability that is aligned with previous research [13, 71–73].

**Fig. 1:**
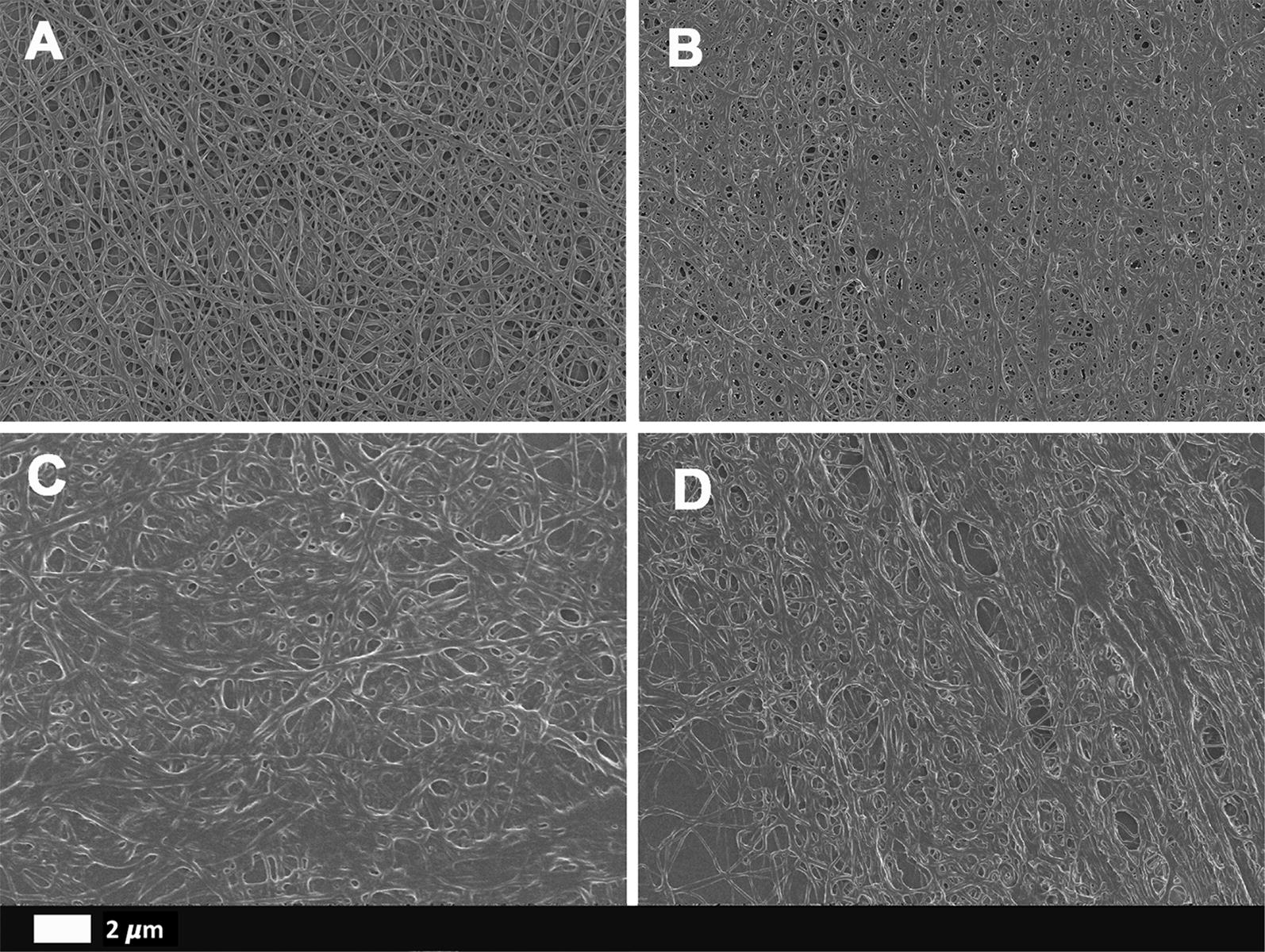
Representative fibrin clot structure of a control (**A**) versus a T2DM individual (**B**–**D**)

To further elucidate what contributes towards the altered TEG results in T2DM individuals (n = 10) compared to controls (n = 10), SEM was used to visualise fibrin ultrastructure. Figure 1 displays representative images of fibrin clots formed from both control and diabetics individuals imaged at 5000- and 10,000-times machine magnification. Through qualitative analysis, it is evident to see that aberrant fibrin clots form in diabetic samples as these clots have fibrin fibres with a greater diameter, multiple fibres meshing together resulting in significantly fewer open spaces between fibres as well as the formation of “dense matted deposits” within the fibrin clots. All of which result in a stronger more stable clots which was concurrently observed in the TEG analysis.

Tables 5 and 6 contain multiplex cytokine and V-Plex vascular injury profile analyses respectively. Note that the vascular injury profile population was a super-set of the cytokine profile population. One can observe many statistically significant differences across the anti-inflammatory, pro-inflammatory and vascular injury marker groups. As with the TEG parameters we see the non-parametric test identifies a (strictly) larger set of parameters as significant, with differences between the models explain by outliers in the control group.

**Table 5:** Anti-inflammatory and pro-inflammatory cytokine profiles and results for healthy and T2DM volunteers

Multiplex cytokine results				
Cytokines (pg mL^−1^)	Healthy (n = 23)	T2DM (n = 25)	Adjusted OR (95% CI)	Mann–Whitney P-value
Anti-inflammatory markers				
IFN-α	1.35 [0.74–1.63]	2.28 [1.94–2.49]	1.03 (0.75, 1.45)	*0.003*
IL-10	2.74 [1.89–5.29]	9.6 [4.62–16.27]	*1.17 (1.05, 1.36)*	*0.0002*
IL-13	2.25 [0.665–7.03]	5.75 [2.56–10.01]	1.03 (0.93, 1.15)	*0.045*
IL-4	7.09 [2.3–20.6]	29.63 [15.3–78.5]	1.01 (1, 1.03)	*0.004*
Pro-inflammatory markers				
E-Selectin	25,885 [19,418–30,622]	24,782 [18,633–35,779]	1 (1, 1)	0.902
GM-CSF	0.001 [0.001–18]	29.18 [15.61–60]	1.02 (1, 1.04)	*0.001*
IFN-γ	4.12 [0.001–12.6]	12.03 [7.1–19.3]	1.02 (0.98, 1.08)	*0.007*
IL-1α	3.49 [2.45–5.2]	11.33 [3.6–24.9]	*1.12 (1.04, 1.23)*	*0.003*
IL-1β	16.47 [11.4–30.3]	48.98 [33.8–76.6]	*1.02 (1, 1.05)*	*0.00005*
IL-12p70	12.03 [6.7–15.4]	22.92 [16.4–30.8]	*1.16 (1.06, 1.3)*	*0.00015*
IL-17A	0.001 [0.001–0.97]	4.11 [1.05–8.47]	1.09 (0.99, 1.24)	*0.0005*
IL-6	8.43 [0.44–21.18]	239.41 [31–1018]	*1 (1, 1.01)*	*0.00005*
IL-8	1.33 [0.001–15.6]	36.32 [3.83–69.5]	1.02 (1, 1.04)	*0.003*
IP-10	18.12 [15.2–27.1]	30.8 [23.0–149.6]	1.01 (1, 1.03)	*0.005*
MCP-1	39.98 [24.8–54.4]	65.3 [43.4–134.2]	*1.04 (1.02, 1.08)*	*0.0002*
MIP-1α	20.94 [4.31–70.7]	63.82 [14.7–108]	1.01 (1, 1.02)	0.083
MIP-1β	39.9 [20.7–424.6]	317.1 [65.2–565.4]	1 (1, 1)	*0.016*
P-Selectin	9080 [6318–12,792]	11,967 [9626–16,135]	1 (1, 1)	*0.03*
sICAM-1	39,140 [27,732–59,821]	47,253 [29,219–58,020]	1 (1, 1)	0.79
TNF-α	86.3 [68.9–121.5]	161.7 [98.6–197.5]	*1.01 (1, 1.03)*	*0.012*

Summary statistics are given as median and [IQR]. Significant differences are shown in italics for both models

**Table 6:** V-Plex vascular injury profiles and results for healthy and T2DM volunteers

V-Plex vascular injury results				
Biomarker (pg mL^−1^)	Healthy (n = 36)	T2DM (n = 39)	Adjusted OR (95% CI)	Mann–Whitney P-value
CRP	1113 [550–2373]	7166 [1749–38,086]	*1.23 (1.09, 1.5)*	*<* *0.0001*
SAA	1532 [807–2876]	8433 [2527–91,382]	*1.02 (1.01, 1.05)*	*<* *0.0001*
sICAM-1	312 [266–357]	367 [300–438]	*447 (4.69,121,580)*	*0.009*
sVCAM-1	387 [327–451]	472 [383–552]	71 (1.55, 9787)	*0.004*

Summary statistics are given as median and [IQR]. Significant differences are shown in italics for both models

Platelet poor plasma lipid levels are shown in Table 7. Healthy controls exhibited significantly higher HDL cholesterol levels compared to T2DM individuals (OR = 0.114). An important consideration is that a subset of our T2DM samples were on lipid-lowering medication (statins) (see Table 3). Conversely, TG concentrations were significantly higher in the diabetic group (OR = 8.34). A trend towards statistical significance was apparent for both TC and LDL, favouring higher levels in healthy individuals relative to diabetes patients.

**Table 7:** Platelet poor plasma blood lipid profile of healthy and T2DM volunteers

Lipid profile results				
Lipogram parameters (mmol L^−1^)	Healthy (n = 23)	T2DM (n = 25)	Adjusted OR (95% CI)	Mann–Whitney P-value
TC	4.19 [3.66–4.79]	3.83 [3.11–4.3]	0.524 (0.223, 1.08)	0.09
HDL	1.74 [1.57–1.91]	1.44 [1.32–1.71]	*0.114 (0.012, 0.64)*	*0.0047*
TG	1.05 [0.81–1.27]	1.29 [1.025–1.65]	*8.34 (1.55, 64.3)*	*0.0139*
LDL	2.14 [1.71–2.36]	1.65 [1.29–1.97]	0.419 (0.128, 1.11)	0.07
Non-HDL	2.51 [2.12–2.88]	2.34 [1.88–2.56]	0.55 (0.159, 1.7)	0.35

Summary statistics are given as median and IQR. Significant differences are shown in italics for both models

**Fig. 2:**
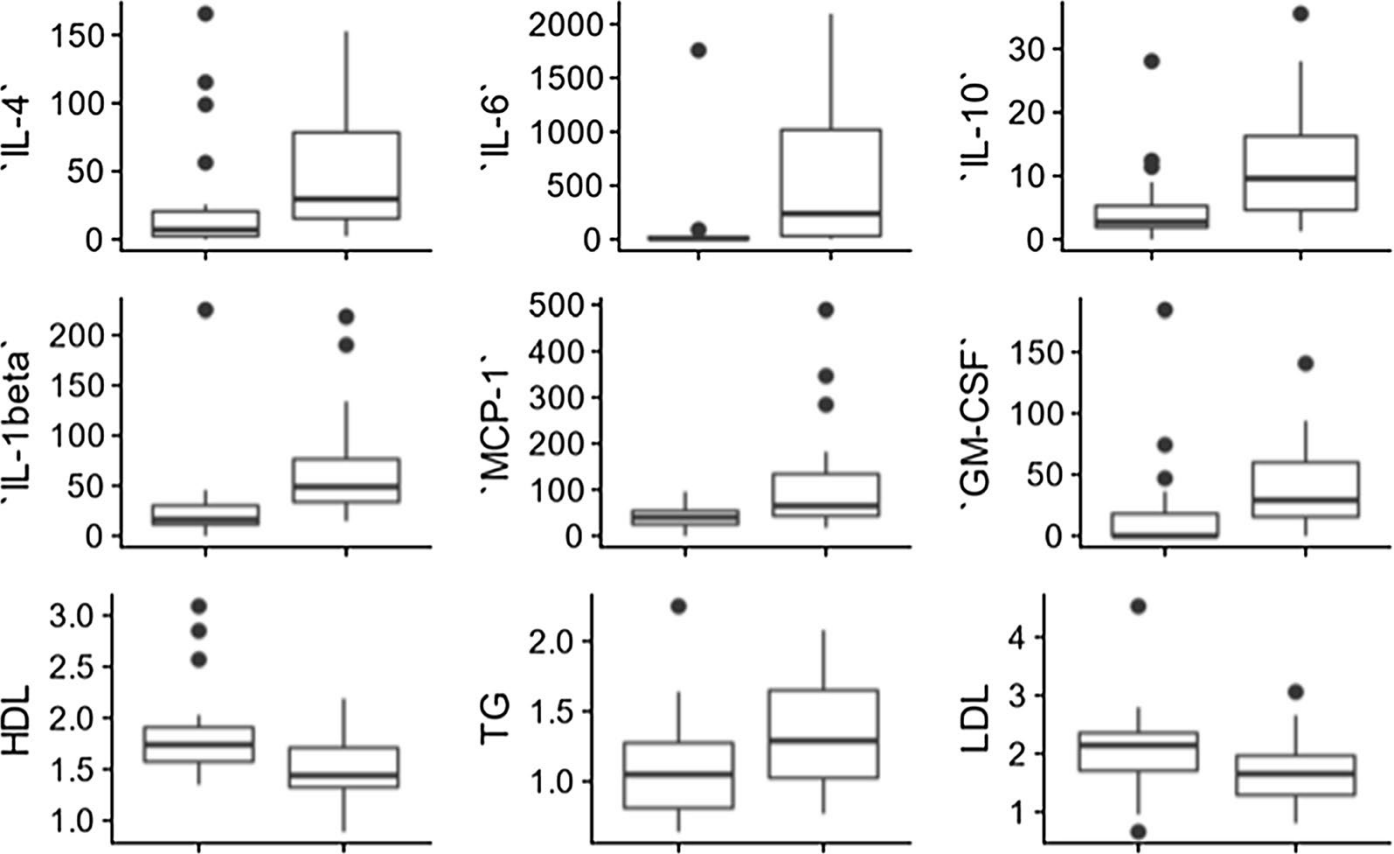
Box and whisker plots for highlighted parameters for healthy (left box) and T2DM populations (right box). Notice the presence of strong outliers, such as the IL-6 healthy group outlier

The differences between the logistic and non-parametric test significance results (also present in the unadjusted fit) are due to the presence of significant outliers, often in the control group, leading to weaker logistic trends. These outliers can be seen in the box and whisker plots in Fig. 2 (IL-6 is a particularly illustrative example). Both models are presented here in order to illustrate both the presence of population differences and regressive trends and finally to highlight consistent parameters across multiple techniques. Future studies on a larger population sizes should attempt to model the outlier behaviour, particularly with regards to outliers in the control population, using a multi-variate model.

**Fig. 3:**
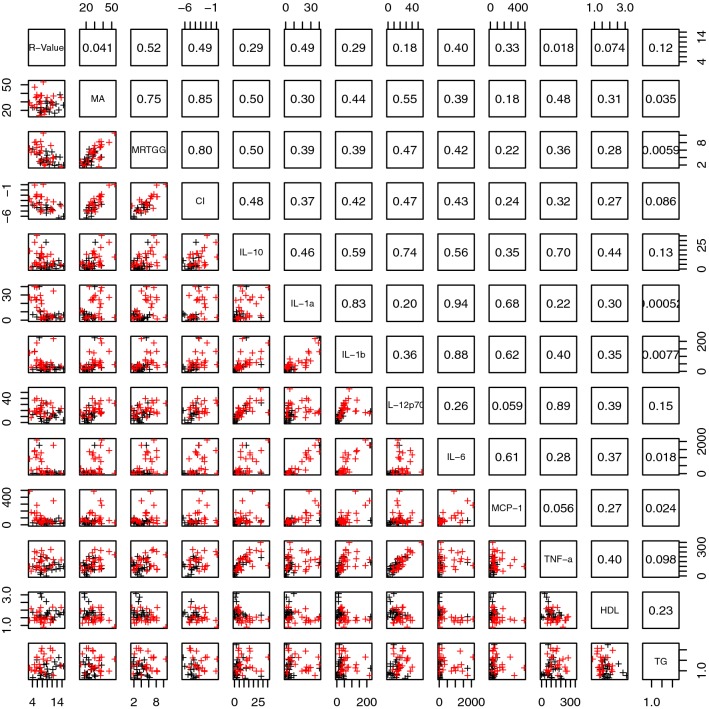
Lattice of cross-plots and correlation values across statistically significant TEG, cytokine and lipid measurements (with groups separated by blue lines). Red represents the T2DM population

**Fig. 4:**
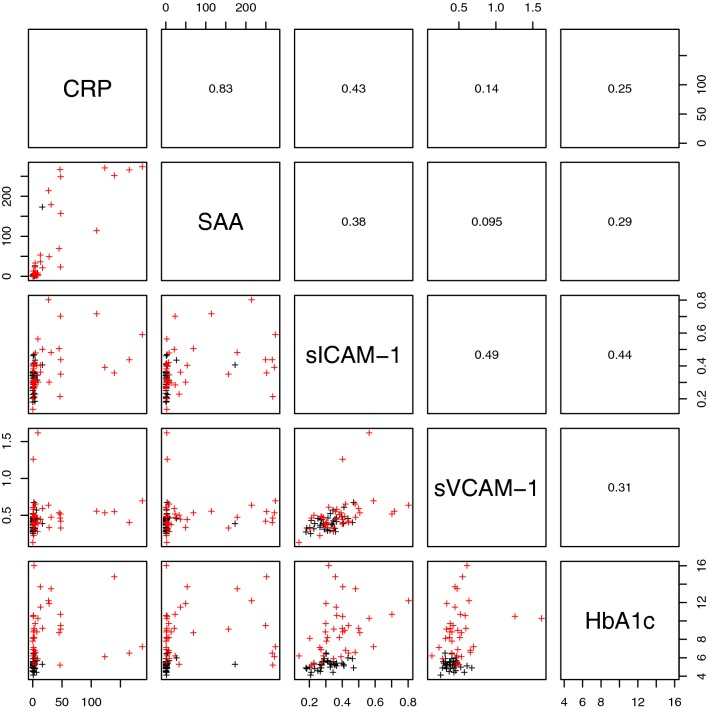
Lattice of cross-plots and correlation values for the vascular injury measurements for the expanded population. Red represents the T2DM population

**Fig. 5:**
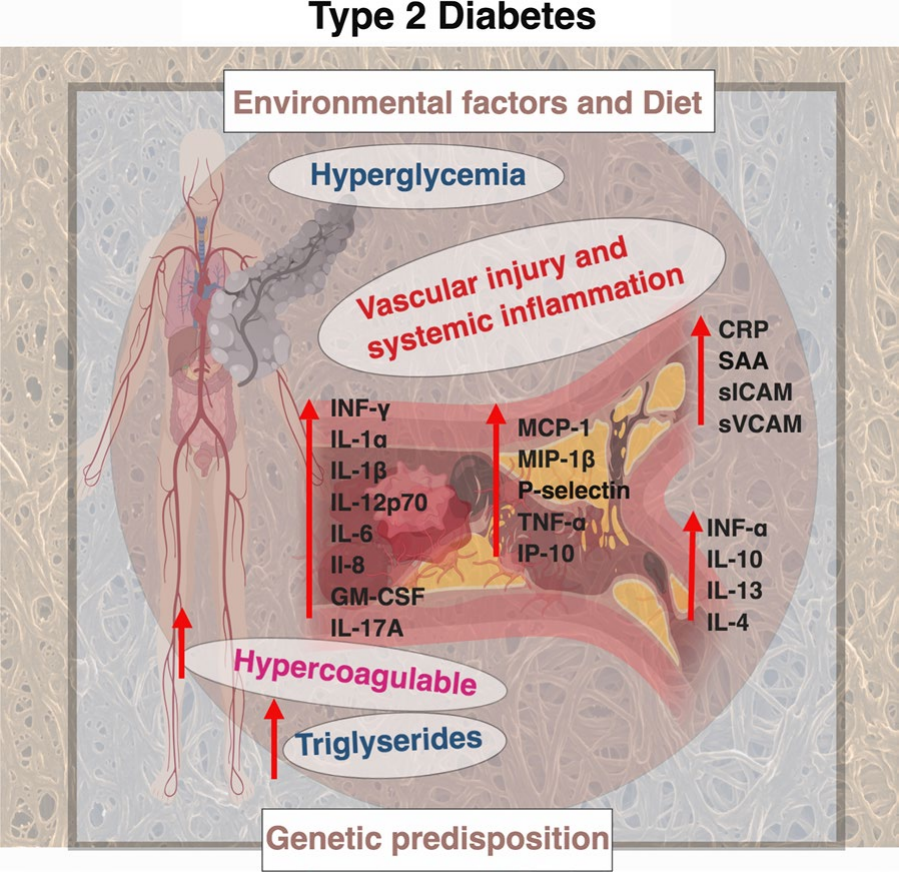
Summary of the circulating biomarkers and hypercoagulation markers of T2DM. Diagram created using BioRender (https://biorender.com/)

Figures 3 and 4 present lattice plots that illustrated the distribution of, and correlations between, the significant parameters. One can observe some strong correlations between parameters both within and between parameter groups. One notable example, as shown in Fig. 5, is the strong correlation between CRP and SAA and sICAM. This correlation is of great importance, as it shows a fundamental relationship between the presence of systemic inflammation, as measured with CRP, and vascular injury, as measured with sICAM. It is known that endothelium overlying atherosclerotic lesions expresses both ICAM-1 (and also VCAM-1 and P-selectin, which are both significantly upregulated in our T2DM sample) [74]. It is also known that ICAM-1, is expressed on endothelium in regions not prone to plaque development.

## Discussion

Analysis of viscoelastic parameters measured by TEG in PPP of control and T2DM individuals showed significant differences between the two groups. Importantly, a decrease clot reaction time (R-value) and decreased clot kinetics (K-value) was observed in the T2DM samples indicating an accelerated initial rate of thrombus generation as well as elevated clot amplification. Furthermore, a statistical trend was seen in the A-angle value indicating that the rate of fibrin build-up and crosslinking is elevated in diabetes, which can be attributed to an elevated thrombin burst in compared to the healthy aged controls. Similarly, a significantly increased maximum clot amplitude (MA) in conjunction with elevated maximal rate of thrombus generation (MRTG) observed in T2DM indicates the formation of a more rigid and stable clot in a significantly accelerated manner. Finally, the significant change in the total thrombus generation (TTG) further indicates the overall elevated strength and stability of the diabetic clots in comparison to that of the healthy controls. The noted aberrant parameters indicate a hypercoagulable state in T2DM which has been previously noted in literature [71–73, 75]. These structural changes in the diabetic fibrin clots were also confirmed using SEM. In future, a comprehensive analysis on fibre structure and thickness measurement could add valuable information, particularly with regards to how fibre structure and thickness may relate to a compromised lytic potential.

The T2DM clots were rich in dense matted deposits, previously shown to correspond to amyloidogenic regions in the fibrin clots [75]. Furthermore, multiple fine fibrin fibres meshes were noticed in the diabetic clots compared to the controls. These findings align with previous evidence. The TEG and SEM results taken together with the circulating biomarker results (Fig. 2) point to a dysregulated circulating molecule presence that might have a direct effect on the development of abnormal clot structure and formation, resulting in a dysregulated coagulation system in T2DM.

Although evidence involving the aberrant role of inflammatory mediators in both communicable and non-communicable disease is indisputable, limited evidence exists regarding the role of inflammatory cytokines in coagulation and fibrinolytic processes in T2DM. Researchers have predominantly investigated relationships through various cell culture models of LPS-induced disease, relating to bacterial and viral infection on hemodynamic processes (see Additional file 1: Tables). Here, we confirm the physiological effect of dysregulated circulating cytokines, and confirm that their presence is accompanied by a hypercoagulable phenotype. These dysregulated circulating cytokines therefore are a direct link between hypercoagulation and inflammation and it manifests in T2DM as a bidirectional relationship between (hyper)coagulation and inflammation. We provide evidence that the majority of inflammatory molecules were elevated in T2DM patients relative to healthy controls a phenomenon that is consistent with established literature (see Additional file 1: Tables S1) particularly with regards to their role in T2DM pathogenesis. Many of the biomarkers studied here, are also known to act as ligands to e.g. platelet, as well as red blood cell (RBC) membrane receptors; examples include e.g. IL-1β, IL-6 and IL-8, where their binding to platelet receptors are known to activate various inflammatory pathways, resulting in platelet hyperactivity, aggregation and spreading, as well as contributing to the circulating inflammatory milieu [76, 77]. Furthermore, the presence of p-selectin, which is a prominent platelet product, is directly indicative of dysregulated platelet aggregation and activation, which can directly drive and actively participate in the development of hypercoagulation [78].

A somewhat salient finding of the present study involves the increased levels of anti-inflammatory cytokines (IFN-α, IL-10, IL-4, IL-13) observed in T2DM patients relative to controls. These molecules are known anti-inflammatory cytokines, but their increased presence in our T2DM sample might be indicative of the very complex interplay between these molecules and their pro-inflammatory counterparts; where the elevated expression of anti-inflammatory cytokines might point to their part as compensatory and counter regulatory molecules, that might aim to limit damage by pro-inflammatory environment. Notably, evidence of elevated anti-inflammatory molecules in disease is by no means an anomaly, and has been observed and interpreted in previous research, however not in the context of T2DM. Consistent with this, a study on IL‐10 and risk of cardiovascular events observed circulating IL‐10 concentrations to be positively associated with pro-inflammatory mediators including CRP and IL‐6 [79]. Additionally, elevated pro-inflammatory cytokines, such as IL-6 and TNF-α, have been observed to accompany anti-inflammatory IL-10 production in certain inflammatory conditions to compensate for inflammation [80]. The physiological role of IL-10 is to limit the inflammatory response by limiting pro-inflammatory cytokine production and suppressing pro-inflammatory T-helper 1 cells natural killer cells and macrophages activity [81]. Thus, it stands to reason that the anti-inflammatory response serves to limit pro-inflammatory activity mediated by elevated pro-inflammatory cytokines, thus elicitinga counter-regulatory role to inflammatory stimuli. We also would like to point out that both our kits, analyzed s-ICAM-1. The Procarta panel is a Luminex system, which uses a fluorescent bead system and a 2-LOG ratio, while the MSD panel uses a 4-LOG ratio. The detection ranges are also as follows: MSD (ICAM-1: 1.94–32,700 pg mL^−1^) and Procarta Luminex (ICAM-1: 151.85–622,000 pg mL^−1^). Our Luminex panel analysis showed a *p* value of 0.79, while our MSD panel analysis showed a P-value of 0.009. Taking this into account, we also suggest that the choice of panels and their sensitivity should be considered if they are used in a clinical setting. Figure 5 shows a summary diagram of the main findings of this paper.

## Conclusion

In conclusion, T2DM is an extremely complex and multifaceted inflammatory disease. These results point to the fact that a personalized approach urgently needs to be followed when planning treatment and tracking the patient heath status after embarking on a treatment regime, and that looking to novel biomarkers might be crucial. Our patient sample were all on various medication interventions, but still their biomarker profiles are significantly different from that of healthy individuals. Diabetes management following a uniform treatment algorithm is often associated with progressive treatment failure and development of diabetic complications [82]. Importantly, a better risk stratification and more precise options for treatment need to be developed and included in clinical practice guidelines [83]. The growing T2DM patient numbers discussed earlier in this paper, suggest that the only way forward is to follow innovative approaches to curb the pandemic we are facing. Perhaps the only option will be to follow an individualized precision medicine approach by using a panel of biomarkers and combining it with e.g. thromboelastography as part of a regular clinical evaluation. By tracking the inflammatory status of T2DM using various biomarkers, before and after embarking on a treatment regime, might allow for a more focussed treatment regime.

## Additional file

**Additional file 1: Table S1.** Summary of inflammatory biomarkers indicating their role in blood clotting (aberrant hypercoagulation and hypofibrinolysis), both in disease and experimental inquisition (*not specific to T2DM). **Table S2.** Inflammatory markers and how their dysregulation contributes towards the development, and/or pathogenesis of T2DM.

## Data Availability

All raw data is available https://1drv.ms/f/s!AgoCOmY3bkKHibUfoOXKPHxT28Ly1w.

## Abbreviations

A angle: alpha angle
ADA: American Diabetes Association
ADP: adenosine diphosphate
AP-1: activator protein 1
CaCl2: calcium chloride
CAF: Central Analytical Facility
CCL3: chemokine (C–C motif) ligand 3
CCL4: chemokine (C–C motif) ligand 4
CLS: crown-like structure
CRP: C-reactive protein
CVD: cardiovascular disease
EDTA: ethylenediaminetetraacetic acid
FFA: free fatty acid
G6P: glucose-6-phosphatase
GLUT: glucose transporter
GM-CSF: granulocyte–macrophage colony-stimulating factor
GPCR: G-protein coupled receptor
GSK-3β: glycogen synthase kinase-3β
HbA1c: glycosylated haemoglobin
HDL: high-density lipoprotein
HMDS: hexamethyldisilazane
HREC: Health Research Ethics Committee
hs-CRP: high sensitivity CRP
HSL: hormone sensitive lipase
HIV: human immunodeficiency virus
IDF: International Diabetes Federation
IFN: interferon
IL: interleukin
iNOS: inducible NO synthase
IP-10: interferon gamma-induced protein 10
IQR: interquartile ranges
IR: insulin resistance
IRS: insulin receptor substrate
JAK-STAT: Janus kinase-Signal Transducer and Activator of Transcription
JNK: c-Jun N-terminal kinase
K: clot kinetics
LDL: low-density lipoprotein
LpL: lipoprotein lipase
LPS: lipopolysaccharide
MA: maximal amplitude
MAPK: mitogen-activated protein kinases
MCP-1: monocyte chemoattractant protein-1
MIP: Macrophage Inflammatory Protein
MRTG: maximum rate of thrombus generation
NF-κB: nuclear factor kappa-light-chain-enhancer of activated B cells
NK: natural killer
NO: nitric oxide
non-HDL: non-high-density lipoprotein
PAI-1: plasminogen activator inhibitor-1
PBS: phosphate-buffered saline
PCA: principal component analysis
PEPCK: phosphoenolpyruvate carboxykinase
PI3-K: phosphatidylinositide-3 kinase
PKA: protein kinase A
PPP: platelet-poor plasma
PRP: platelet-rich plasma
RBC: red blood cell
ROS: reactive oxygen species
RTK: receptor tyrosine kinase
R-value: clot reaction time
SEM: scanning electron microscopy
SEMSDA: Society for Endocrinology, Metabolism and Diabetes of South Africa
SHIP1: Src homology 2 (SH2) domain containing inositol polyphosphate 5-phosphatase 1
sICAM-1: soluble intercellular adhesion molecule-1
SOCS: suppressor of cytokine signaling
sP-selectin: soluble P-selectin
SST: serum separating tubes
T2DM: type 2 diabetes mellitus
TC: total cholesterol
TEG: thromboelastography
TF: tissue factor
TG: triglyceride
TLR: toll-like receptor
TMRTG: time to maximum rate of thrombus generation
TNF-α: tumor necrosis factor-alpha
tPA: tissue-type plasminogen activator
TTG: total thrombus generation
TXA2: thromboxane A2
VLDL: very low-density lipoprotein
vWF: von Willebrand Factor
WB: whole blood
