# Supplementary material for: Inflammatory cytokines in type 2 diabetes mellitus as facilitators of hypercoagulation and abnormal clot formation
Source: Cardiovasc Diabetol. 2019 Jun 4;18:72. doi: 10.1186/s12933-019-0870-9 (PMC6549308; doi:10.1186/s12933-019-0870-9)
Supplement: Supplementary file 1 — Additional file 1: Table S1. Summary of inflammatory biomarkers indicating their role in blood clotting (aberrant hypercoagulation and hypofibrinolysis), both in disease and experimental inquisition (*not specific to T2DM). Table S2. Inflammatory markers and how their dysregulation contributes towards the development, and/or pathogenesis of T2DM. [file 12933_2019_870_MOESM1_ESM.docx]

**Table S1**: Summary of inflammatory biomarkers indicating their role in blood clotting (aberrant hypercoagulation and hypofibrinolysis), both in disease and experimental inquisition **(*not specific to T2DM).**

| **Biomarker** | ***Role in hypercoagulation** |
| --- | --- |
| **IFN-α** | Exogenous interferon alpha (IFN-α) administration induces fibrinolysis-stimulating tissue-type plasminogen activator (tPA) expression in in microvascular endothelial cells via Signal transducer and activator of transcription 1 (STAT1) activation (Strandin et al., 2016).  IFN-α have been recognized to enhance tPA secretion by macrophages in culture (Hovi et al., 1981, Jones et al., 1982).  Human studies involving IFN-α therapy has also been observed to augment tPA expression and fibrinolysis (Gliozzi et al., 2013, Gokmen et al., 2011).  In contradiction, Jia *et al*., demonstrated that IFN-α treatment reduces endothelial cells fibrinolytic activity through the greater upregulation of Plasminogen activator inhibitor-1 (PAI-1) than tPA. PAI-1 swiftly binds and inactivates tPA, thus mediating IFN-impaired fibrinolysis (Jia et al., 2018). |
| **IL-10** | IL-10 attenuates the tissue factor activity of fibrin-adherent monocytes *in vitro* (Veltrop et al., 2001) and has been demonstrated to further downregulate tissue factor (TF) expression and activity in cultured human monocytes (Ernofsson et al., 1996, Kamimura et al., 2005, Lindmark et al., 1998).  Activated protein C, an anticoagulant serine protease protein, upregulated IL-10 production and mRNA synthesis in lipopolysaccharides (LPS)-stimulated monocytes, via p38 mitogen-activated protein kinases (MAPK) activation (Toltl et al., 2008).  IL-10 was demonstrated to effectively suppress IL-1α/β-induced procoagulant activity by human monocytes i*n vitro* (Osnes et al., 1996).  IL-10 attenuates thrombin generation kinetics of LPS-stimulated monocytes and reduces both monocyte TF expression and the release of active TF-bound microparticles (Poitevin et al., 2007). |
| **IL-13** | IL-13 suppresses IL-1β-, TNF-α- and LPS-induced TF induction in cultured endothelial cells (Herbert et al., 1993).  IL-13 was demonstrated to effectively suppress IL-1α/β-induced procoagulant activity by human monocytes i*n vitro* (Osnes et al., 1996).  IL-13 down-regulates LPS-induced TF expression in human monocytes (Ernofsson et al., 1996).  IL‐4 down‐regulates exaggerated IL‐6-stimulated fibrinogen secretion *in vitro*, this effect being more pronounced prior to IL-6 addition (Vasse et al., 1996). |
| **IL-4** | IL-4 attenuates proinflammatory-induced TF expression by affected endothelial cells and monocytes, thus counteracting aberrant procoagulant processes (Herbert et al., 1992).  IL-4 inhibits thrombomodulin expression by endothelial cells, therefore abrogating its anticoagulant activity (Herbert et al., 1992, Herbert et al., 1993).  IL-4 suppressors IL-1β-, TNF-α- and LPS-induced TF induction in cultured endothelial cells (Herbert et al., 1993).  IL-4 down-regulates LPS-induced TF expression in human monocytes (Ernofsson et al., 1996).  IL-4 was demonstrated to effectively suppress IL-1α/β-induced procoagulant activity by human monocytes i*n vitro* (Osnes et al., 1996).  IL‐4 down‐regulates exaggerated IL‐6-stimulated fibrinogen secretion *in vitro*, this effect being more pronounced prior to IL-6 addition (Vasse et al., 1996). |
| **E-Selectin** | Low concentrations of thrombin inhibit E-selectin expression by endothelial cells (Bae et al., 2009).  Thrombin activates endothelial cells and through this mode of activation enhances E-selectin expression (Kaplanski et al., 1997).  E-selectin-linked signalling cascade induces tissue factor production by endothelial cells (Schmid et al., 1995).  E-selectin expressed on activated endothelial cells binds to activated platelets thus potentiating thrombus formation (Kappelmayer and Nagy, 2017). |
| **GM-CSF** | Anticoagulant, protein C, was found to be reduced in GM-CSF recipients (Bonig et al., 2001)  GM-CSF increased the levels of urokinase-type plasminogen activator (u-PA) activity and of u-PA mRNA in purified human monocytes (Hart et al., 1991)  co-stimulation of monocytes with GM-CSF and IFN-y enhanced t-PA activity, however independent culturing of either cytokine induced detectable t-PA activity (Hart et al., 1991)  GM-CSF enhances constitutive levels of PAI-1 and PAI-2, thrombogenic proteins that inhibit fibrinolysis, in cultured human monocytes (Hamilton et al., 1993)  TF expression is upregulated by GM-CSF cultured macrophages (Yamaguchi et al., 2016) |
| **IFN-γ** | IFN-γ induces procoagulant activity in macrophages (Schwager and Jungi, 1994).  Exogenous IFN-γ administration was unexpectedly found to induce tPA expression in microvascular endothelial cells via STAT1 activation and binding to tPA enhancer elements, thus enhancing fibrinolytic processes (Strandin et al., 2016).  IFN-γ-treatment of macrophages resulted in amplified tPA expression and augmented plasmin generation, via Janus kinase (JAK)-Signal Transducer and Activator of Transcription (STAT)-dependent signalling pathways (Gliozzi et al., 2013).  IFN-γ exerts a counter regulatory role to TNF-α by supressing fibrin deposition, acting via STAT1 and IFN-γ receptors on radio resistant cells (Mullarky et al., 2006). |
| **IL-1α** | IL-1α administration induces thrombin formation in baboons after bacterial infection (Jansen et al., 1995). |
| **IL-1β** | IL-1β induces procoagulant activity in macrophage and endothelial cells (Schwager and Jungi, 1994).  IL-1β enhances TF expression and activity by endothelial cells and monocytes (Herbert et al., 1992).  IL-1β induces IL-6 signalling pathways resulting in pro-coagulant fibrinogen synthesis (Duan et al., 2010, Yang et al., 2013)IL-1 may indirectly regulate coagulation via inducing neutrophil serine protease activation and subsequently activating TF and factor XII-dependent coagulation pathways which stimulate thrombus formation (Massberg et al., 2010).  IL-1β induces clotting factor VII synthesis in monocytes (Carlsen et al., 1988)IL-1β downregulates thrombomodulin expression thus hindering anticoagulant pathways, particularly via attenuated anticoagulant protein C activation (Bester and Pretorius, 2016).  IL-1β triggers platelet hyperactivation and induces faster clot formation (hypercoagulation) (Bester et al., 2018, Bester and Pretorius, 2016).  IL-1β has been demonstrated by Suharti *et al.,* to be positively associated with tPA (Suharti et al., 2002). |
| **IL-12p70** | Addition of IL-12 to whole blood resulted in red blood cell (RBC) agglutination and platelet hyperactivation, further compounded by thrombogenic interactions between activated platelets and agglutinated RBCs (Page et al., 2018).  IL-12 administration to human whole blood favours hypercoagulable clot formation, as measured by TEG parameters (Page et al., 2018).  Thrombin, a procoagulant serine protease, supresses IL-12 release and expression of IL-12 and enhances IL-10 production in human peripheral blood mononuclear cells (Naldini et al., 2003) |
| **IL-17A** | IL-17 independently, and in combination with TNFα, enhances TF gene expression (Hot et al., 2012).  IL-17 attenuates anticoagulant thrombomodulin expression by endothelial cells (Hot et al., 2012).  IL-17 enhances platelet agglutination (Hot et al., 2012). |
| **IL-6** | IL-6 signalling mediates Stat3 phosphorylation which subsequently promotes fibrinogen gene expression (Duan et al., 2010).  IL-6 upregulates TF thus initiating coagulation (Bester and Pretorius, 2016).  IL-6 triggers platelet hyperactivation (Bester and Pretorius, 2016). |
| **IL-8** | IL-8 triggers platelet hyperactivation thus promoting procoagulant activity (Bester and Pretorius, 2016, Regnault et al., 2003).  IL-8 administration accelerates hypercoagulation (faster clot formation) in whole blood and enhanced fibrin fibre cross-linking (stronger clot) (Bester et al., 2018).  Coagulation factor Xa stimulates IL-8 and MCP-1 expression in endothelial cells and leukocytes (Busch et al., 2005). |
| **IP-10** | Research regarding the haemostatic properties of IP-10 is limited |
| **MIP-1α (CCL3)** | TF is associated with the increased release of the monocyte chemoattractant molecule MIP-1α antecedent to *in vitro* splenocyte stimulation (Bokarewa et al., 2002) |
| **MIP-1β (CCL4)** | Fibrinogen exposure stimulates MIP-1β upregulation in macrophage cell-lines (Smiley et al., 2001) |
| **MCP-1** | Coagulation factor Xa stimulates MCP-1 expression in endothelial cells and leukocytes (Busch et al., 2005).  Thrombin stimulates MCP-1 expression by circulating human monocytes (Ernofsson and Siegbahn, 1996).  MCP-1 induces TF expression by human monocytes (Ernofsson and Siegbahn, 1996). |
| **P-Selectin** | P-selectin induces platelet and leukocyte rolling on activated endothelium, thus mediating platelet-leukocyte interaction (Frenette et al., 1995).  Agonists of P-selectin’s plasma membrane expression on platelets and endothelial cells includes the coagulation protein, thrombin (Crovello et al., 1993).  Leukocyte-binding properties of P-selectin expressed on platelets mediates the accumulation of leukocytes into thrombi *in vivo*, thus enhancing thrombus growth. Leukocytes incorporated into growing thrombus also promoted fibrin deposition.(Palabrica et al., 1992).  Potentially involved in the clearance of thrombi by macrophages, as is evident by persistent thrombi in P-selectin-deficient mice (Subramaniam et al., 1996).  Activated platelets within thrombi express P-selectin thus facilitating leukocytes recruitment and promote leukocyte-mediated fibrin deposition within thrombi (Palabrica et al., 1992).  P-selectin expressed on activated platelets also upregulates the expression, and pro-coagulant activity, of TF on monocytes (Celi et al., 1994).  P-selectin is proteolytically shed after platelet activation, therefore high levels of soluble P-selectin reflect augmented platelet activation (Michelson et al., 1996).  P-selectin stimulates leukocyte-derived microparticle generation thus promoting microparticle-mediated procoagulant activity (Andre et al., 2000) such as enhanced prothrombinase expression (Satta et al., 1994), elevated TF expression by endothelial cells (Mesri and Altieri, 1999).  P-selectin is also expressed on platelet-derived microparticles, generated after platelet activation (Zeiger et al., 2000). |
| **sICAM-1** | Low concentrations of thrombin inhibit intercellular adhesion molecule-1 (ICAM-1) expression by endothelial cells (Bae et al., 2009).  Thrombin induces ICAM-1 expression on endothelial cells (Kaplanski et al., 1998), via NF-κB activation (Rahman et al., 1999). |
| **TNF-α** | IL-1β enhanced TF expression and activity by endothelial cells and monocytes (Herbert et al., 1992).  TNF-α exhibits anti-fibrinolytic activity by inducing the synthesis and secretion of PAI-1 (Pandey et al., 2005)and down-regulating tissue-type plasminogen activator (t-PA) expression in endothelial cells (Massignon et al., 1994).  TNF-α enhances TF procoagulant activity and suppresses Protein C anticoagulant function (Nawroth and Stern, 1986).  TNF-α exposure facilitates aberrant platelet aggregation and activation (Hot et al., 2012, Manfredi et al., 2016, Page et al., 2018).  Additionally, TNF-α enhances the synthesis and surface expression of tissue factor, by downregulating thrombomodulin secretion (Scarpati and Sadler, 1989).  TNF-α exerts a counter regulatory role to IFN-γ by promoting fibrin deposition via type 1 TNF-α receptors (Mullarky et al., 2006).  TNF-α has been implicated in enhancing fibrinolytic activity through its significant association with D-dimer, a fibrin degradation product and marker of fibrinolysis activation (Suharti et al., 2002) |

**Table S2**: Inflammatory markers and how their dysregulation contributes towards the development, and/or pathogenesis of T2DM.

|  | **Role in Type II Diabetes Mellitus pathogenesis** |
| --- | --- |
| **IFN-α** | Research limited to type 1 diabetes mellitus, however, IFN-α has been demonstrated to delay/inhibit the maturation of monocytes to macrophages *in vitro*, a phenomenon that may be implicated in IFN- α-induced immunosuppression (Lee and Epstein, 1980). |
| **IL-10** | Low circulating levels of IL-10 and low IL-10 production capacity have been reported in T2DM (van Exel et al., 2002; Yaghini et al., 2011).  **Hypo-responsive IL10 anti-inflammatory function contributes to inflammatory response in T2DM** (Barry et al., 2016, Murray, 2006)  Reduced anti-inflammatory monocyte responsiveness to IL-10 in the presence of hyperglycaemic conditions is not mediated by IL10 receptor downregulation but via supressed IL-10 phosphorylation of STAT-3 and downstream IL-10 signal transduction in monocytes (Barry et al., 2016, Murray, 2006).  Immunosuppressive effects of IL-10 involves both inhibition of inflammatory cytokine synthesis (e.g., TNF-α, IL-6) and their biological activities on target cells via a Src homology 2 (SH2) domain containing inositol polyphosphate 5-phosphatase 1 (SHIP1)-dependent pathway (Chan et al., 2012), thus limiting the production and action of pro-inflammatory molecules.  Systemically, IL-10 inhibits pro-inflammatory M1 polarisation and activation of macrophages (Moore et al., 2001)and activates 5′AMP-activated protein kinase (AMPK) pathways in macrophages thus prompting anti-inflammatory (M2 phenotype) macrophage polarization (Sag et al., 2008, Zhu et al., 2015).  **Protective role of anti-inflammatory IL‐10 in in T2DM**  IL-10 enhances glucose uptake in adipocytes and protects against TNF-α induced IR (Lumeng et al., 2007).  IL-10 upregulates the tyrosine kinase activity of the insulin receptor, including enhanced insulin receptor substrate-1 (IRS-1), and Akt activity in skeletal muscle (Hong et al., 2009).  IL-10 has been positively associated with circulating IL-10 levels and whole-body insulin sensitivity in healthy individuals (Straczkowski et al., 2005). |
| **IL-13** | **Compensatory/protective role of IL-13 in T2DM against insulin resistance and β-cell failure**  IL-13 *in vitro* enhances insulin secretion by pancreatic β-cells of humans and rats (Rutti et al., 2016)prompting a compensatory hyper-insulinaemic response to oppose hyperglycaemia and attenuated insulin sensitivity and facilitating improved β-cells functioning.  IL-13 gene ablation has been shown to diminish IRS-1 and Akt phosphorylation in liver, adipose tissue, and skeletal muscle of mice (Stanya et al., 2013) thus attenuating glucose uptake in the aforementioned insulin target tissues (i.e. Reduced insulin sensitivity).  IL-13 induces IRS-1 and Akt phosphorylation via STAT3 and STAT6 activation and thus protects against IR (Stanya et al., 2013).  Notably, IL-13 positively regulates β-cell survival via the IRS-2/Akt pathway (Rutti et al., 2016), resulting in IL-13 protection against β-cell death, specifically against IL-1β-induced death (Rutti et al., 2016).  IL-13 acts as a myokine that promotes skeletal muscle glucose metabolism by enhancing glycogen synthesis (Jiang et al., 2013).  IL-13 induces alternative activation towards the anti-inflammatory M2 macrophage phenotype in adipose tissue, resulting in enhanced insulin sensitivity in mice (Kang et al., 2008) and has a suppressive effect on M1 macrophage activity, and therefore, the reduced IL-13 levels may promote the release of proinflammatory cytokines (Olefsky and Glass, 2010).  Constitutive IL-13 expression is required for normal postprandial suppression of hepatic glucose production in mice (Stanya et al., 2013).  **IL-13 overproduction enhances IL-13-mediated T2DM pathogenicity**  Strong, positive associations between IL-13 and elevated triglyceride (TG) levels (hypertriglyceridemia) by Martínez-Reyes et al. proposes a role of IL-13 in the upregulation of lipolysis in T2DM (Martinez-Reyes et al., 2018).  IL-13 mediates its effects on hepatic glucose production via STAT3 phosphorylation (p-STAT3) (Stanya et al., 2013). IL-13 binding within hepatocytes facilitates p-STAT3 accumulation and suppresses gluconeogenic gene expression, such as phosphoenolpyruvate carboxykinase (PEPCK) and glucose-6-phosphatase (G6P), resulting in reduced hepatocyte glucose production (Akaiwa et al., 2001, Stanya et al., 2013).  Excessive IL-13 production is potentially detrimental to liver tissue; IL-13 is proposed to directly activate hepatic stellate cells to a fibrogenic phenotype (Sugimoto et al., 2005). Consequently, IL-13 is strongly associated with steatohepatitis and liver fibrosis, hallmarks of non-alcoholic fatty liver disease (Weng et al., 2009). |
| **IL-4** | **Protective role of IL-4 in insulin target tissues affected by T2DM**  IL-4 promotes glucose tolerance and insulin sensitivity in muscle and fat cells via upregulated Akt phosphorylation and attenuated Glycogen synthase kinase-3β (GSK-3β) activity (Chang et al., 2012).  IL-4 mediates a protective effect against pancreatic β-cell loss via IRS-2/PI3K/Akt and JAK3/STAT6 signalling resulting in cytoprotection and anti-apoptotic signals (Kaminski et al., 2009).  Evidently, chronic insulin and high glucose conditions, reminiscent of T2DM conditions, synergistically inhibits IL-4-dependent activation of PI3-kinase in macrophages and inhibits IL-4-mediated anti-inflammatory M2 macrophage polarisation (Hartman et al., 2004).  **Suggestive pathological and diabetogenic action of IL-4 in adipose tissue**  IL-4 exhibits anti-lipogenic capability via protein kinase A (PKA) pathways that facilitate upregulated hormone sensitive lipase (HSL) activity, thus promoting lipolysis and free fatty acid (FFA) release (Tsao et al., 2014).  IL-4 inhibits adipogenesis via STAT6 activation in adipocytes (Tsao et al., 2014). |
| **E-Selectin** | Elevated E-Selectin levels evident in T2DM (Ceriello et al., 2004, Elhadd et al., 2004, Kistorp et al., 2008).  Insulin stimulates nitric oxide (NO) production within endothelial cells, precipitating in the overexpression of cellular adhesion molecules, such as E-selectin (Kourembanas et al., 1993, Steinberg et al., 1994). |
| **GM-CSF** | Decreased granulocyte-macrophage colony-stimulating factor (GM-CSF) levels is associated with increased food intake and may in part mediate the pathological T2DM hallmark of polyphagia (Reed et al., 2005).  GM-CSF can act as a strong chemoattractant for monocytes (O'Brien et al., 1998), and is a macrophage recruitment and activation factor in adipose tissue, enhancing inflammation (Kim et al., 2008). |
| **IFN-γ** | **Enhanced macrophage-mediated pathology by IFN-γ in T2DM**  IFN-*γ* enhances macrophage infiltration in adipose tissue (Rocha et al., 2008, Zhang et al., 2011).  IFN-*γ* facilitates endothelial dysfunction through increased macrophage accumulation and vascular wall MCP-1 expression (Zhang et al., 2011).  Enhanced IFN-*γ* expression facilitates necrotic crown-like structure (CLS) formation in adipose tissue via increased T-lymphocyte/macrophage infiltration (McGillicuddy et al., 2009).  **IFN-γ mediates T2DM pathophysiology**  IFN-γ treatment attenuated adipocyte IRS-1 and glucose transporter (GLUT)-4 protein levels resulting in reduced glucose uptake and AKT phosphorylation (i.e. insulin resistance) (McGillicuddy et al., 2009).  Long-term IFN-γ treatment reduced lipid storage in human adipocytes via the mRNA suppression of adipogenic genes such as fatty acid synthase, lipoprotein lipase (LpL) and adiponectin (McGillicuddy et al., 2009).  *In vitro* administration of IFN-γ facilitates pancreatic β-cell destruction (Rabinovitch and Suarez-Pinzon, 1998). |
| **IL-1α** | **Diabetogenic action of elevated IL-1 facilitates T2DM pathology**  Osborne *et al*., demonstrated that neutralisation of IL-1β alone, without inhibiting IL-1α action, significantly improved the glycaemic control of pancreatic tissue. This is suggestive that IL-1β is the key cytotoxic mediator of impaired function and destruction of pancreatic beta cells in both type 1 and 2 diabetes (Osborn et al., 2008b).  IL-1α directly inhibits lipid accumulation in adipocytes by suppressing LpL expression in adipocytes (Fried et al., 1993).  IL-1β downregulates [GLUT2](https://www.sciencedirect.com/topics/medicine-and-dentistry/glucose-transporter-2) expression in insulin-target tissues, such as the liver and [pancreatic islets](https://www.sciencedirect.com/topics/medicine-and-dentistry/pancreatic-islet), inhibiting glucose uptake (Metzger et al., 2004, Park et al., 1999).  IL-1β impairs insulin-induced glucose uptake by adipocytes via the down regulation of IRS-1 and subsequently, reduced GLUT4 translocation (Jager et al., 2007).  Excessive IL-1 concentrations suppress proinsulin production and induce β-cell apoptosis (Mandrup-Poulsen et al., 1993).  IL-1β-nuclear factor kappa-light-chain-enhancer of activated B cells (NF-κB)-mediated glucotoxicity in pancreatic β-cells; high glucose (hyperglycaemia) triggers IL-1 β production by β-cells thus triggering “autocrine apoptosis” via IL-1β/NF-κB activation and upregulated Fas signalling triggering (Donath et al., 2003, Maedler et al., 2002).  Two-fold effect of IL-1 on β-cells; low concentrations of IL-1β stimulate pancreatic β-cell proliferation and insulin secretion (Maedler et al., 2006), whereas elevates IL-1β levels impairs insulin secretion and facilitates pancreatic β-cell apoptosis (Kiely et al., 2007, Osborn et al., 2008a). |
| **IL-1β** |  |
| **IL-12p70** | **IL-12, a product and arbitrator of T2DM and associated cardiovascular complications**  Hyperglycaemia stimulates macrophage IL-12 gene expression in diabetic animal models (Wen et al., 2006).  IL-12 expression by β-cells induces β-cell apoptosis via IL-12/STAT4 signalling and enhanced β-cell expression of IFN-γ (Taylor-Fishwick et al., 2013, Weaver et al., 2015).  **Pro-atherosclerotic role of IL-12 in T2DM**  IL-12 contributes to the process of atherosclerotic plaque formation and probably accelerates the development of macrovascular complications in type 2 diabetics (Hauer et al., 2005, Uyemura et al., 1996).  Elevated IL-12 levels stimulate T lymphocytes and natural killer (NK) cell proliferation and migration to atherosclerotic plaques (Zhang et al., 2006) and favours the development of a pro-atherosclerotic Th1 cell phenotype (Frostegard et al., 1999, Manetti et al., 1993).  IL-12 inhibits angiogenesis in a type 2 diabetic murine model (Ali et al., 2017).  IL-12 enhances the release of IFN-γ (Fei et al., 2003), which has aggravating effects on atherosclerosis. (Gupta et al., 1997). |
| **IL-17A** | **Hepatotoxic effects of IL-17A in T2DM**  IL-17A activated JAK2/STAT3/ suppressor of cytokine signaling (SOCS)-1 signalling pathway facilitates hepatic insulin resistance (Zhang et al., 2012).  It has also been established that STAT-3 signalling is involved in beta-cell apoptosis (Oh et al., 2011) and that STAT3 activation promotes insulin resistance within hepatocytes and skeletal muscle (Kim et al., 2013, Serrano-Marco et al., 2012).  IL-17A/STAT3 signalling cascade has also been implicated in fibrosis induction and apoptosis in hepatocytes (Meng et al., 2012).  Additionally, STAT3 also suppresses the gluconeogenic gene expression in the liver (Inoue et al., 2006).  IL-17 stimulates inducible NO synthase (iNOS) expression/activation via P38-MAPK/activator protein 1 (AP-1)/iNOS signalling pathway and resultantly enhances NO-dependent toxic damage of pancreatic β-cells (Miljkovic et al., 2005). |
| **IL-6** | **IL-6 mediates insulin resistance various target-tissues**  IL-6 induces insulin resistance via attenuated GLUT4 and IRS-1 expression and activity in skeletal muscle (Rotter et al., 2003).  IL-6/SOCS-3 signalling in adipocytes upon insulin stimulation acts as a negative regulator of insulin signalling by binding to and ubiquitinating IRS-1 directing such signalling intermediates for proteasomal degradation (Emanuelli et al., 2000).  IL-6 attenuates insulin signalling in hepatocytes via inhibition of insulin receptor autophosphorylation and tyrosine phosphorylation of IRS-1 resulting in decreased glycogen storage due to decreased gluconeogenesis and increased glycogenolysis. (i.e. reduced insulin sensitivity) (Klover et al., 2003, Kristiansen and Mandrup-Poulsen, 2005, Senn et al., 2002, Senn et al., 2003).  **Anti-lipogenic effects of IL-6 in T2DM**  IL-6 enhances systemic fatty acid oxidation and lipolysis T2DM (Petersen et al., 2005, van Hall et al., 2003), thus increasing FFAs in circulation.  Additionally, IL-6 also down-regulates LpL expression in mature adipocytes thus inhibiting lipid accumulation in adipocytes (Greenberg et al., 1992).  IL-6 stimulates ROS production and reduces NO bioavailability (Zhang and Zhang, 2009). |
| **IL-8** | Aberrant stimulation of hepatocytes by FFAs induces hepatocyte IL-8 production via NF-kB activation and c-Jun N-terminal kinase (JNK) pathways (Joshi-Barve et al., 2003).  IL-8 produces insulin resistance via the inhibition of insulin-induced Akt phosphorylation in human adipocytes (Kobashi et al., 2009).  Excess IL-8 secretion by skeletal muscle under T2DM conditions reduces myotube formation and capillary density resulting in **c**achexia, hypoxia and limiting substrate availability, such as glucose (Amir Levy et al., 2015).  IL-8 further exacerbates muscle glucose disposal via reduced myotube GLUT4 translocation (Amir Levy et al., 2015). |
| **IP-10** | Interferon gamma-induced protein 10 (IP-10) acts as a chemoattractant for proinflammatory Th1 lymphocytes (Gasperini et al., 1999).  IP-10 is produced and secreted by cultured human islets of patients with T2DM, localised in β cells (Schulthess et al., 2009).  Schulthess *et a*l. found that IP-10 elicits islet β-cell apoptosis via toll-like receptor (TLR)-4/Akt signalling and prolonged activation of JNK in patients with T2DM (Schulthess et al., 2009). |
| **MCP-1** | **MCP-1 mediates and enhances T2DM pathology in insulin target tissues**  MCP-1 release from β cells facilitate the recruitment and infiltration of monocytes into pancreatic islets thus contributing to the loss/dysfunction of insulin-producing β cells (Boni-Schnetzler et al., 2008, Piemonti et al., 2002).  MCP-1 blunts insulin-stimulated glucose uptake in cultured adipocytes thus promoting insulin resistance (Gerhardt et al., 2001, Sartipy and Loskutoff, 2003).  MCP-1 mediates aberrant lipid metabolism by down-regulating LpL expression in adipocytes, thus inhibiting lipid accumulation in adipocytes (Gerhardt et al., 2001, Sartipy and Loskutoff, 2003).  MCP-1 stimulates reactive oxygen species (ROS) production and reduce NO bioavailability (Zhang and Zhang, 2009).  **MCP-1 accelerates the rate of atherosclerosis**.  MCP-1 attracts and initiates migration of monocytes into the vascular wall thus facilitating foam cell development (Piemonti et al., 2009).  High glucose treatment (Hyperglycaemia) enhances MCP-1 release from smooth muscle cells (SMC) thus facilitating increased monocyte-SMC adhesion via MAPK/NFκB activation (Dragomir et al., 2008) and induces cardiomyocyte death via elevated autophagy, endoplasmic reticulum (ER) stress and reactive oxygen species (ROS) production (Younce et al., 2010).  Oxidised lipoproteins (specifically low-density lipoprotein (LDL)) isolated from type 2 diabetic subjects stimulated mRNA gene expression of MCP-1 in endothelial cells via the NFκB pathway (Takahara et al., 1997). |
| **MIP-1α (CCL3)** | Macrophage Inflammatory Protein (MIP)-1α, also known as chemokine (C-C motif) ligand 3 (CCL3), research is limited to type 1 diabetes mellitus.  Very low-density lipoprotein (VLDL) incubation induces CCL3 expression (Saraswathi and Hasty, 2006). |
| **MIP-1β (CCL4)** | Limited research in T2DM regarding chemokine (C-C motif) ligand 4 (CCL4) function.  CCL4 is a major macrophage attractant; CCL4 may indirectly facilitate β-cell death by macrophages (potential pathogenesis in T1DM and T2DM) (Benoist and Mathis, 1997, DeVries et al., 1999).  Insulin supresses CCL4 expression in mononuclear cells (Dandona et al., 2001).  High glucose conditions mediate augmented CCL4 secretion by macrophages (Chen et al., 2011). |
| **P-Selectin** | P-selectin is elevated in T2DM (Gokulakrishnan et al., 2006, Urbanovych et al., 2016, Woollard et al., 2014).  **P-Selectin, a product and potential marker of T2DM pathophysiology**  Hyperglycaemia elevates plasma soluble P-selectin in type 2 diabetic patients (Yngen et al., 2001).  Endothelial dysfunction, a component of insulin resistance in type 2 diabetes mellitus, is characterized by the hypersecretion of Р-selectin by platelet membranes indicating increased agglutination and platelet aggregation (i.e. platelet hyperactivity) (Urbanovych et al., 2016, Woollard et al., 2014). |
| **sICAM-1** | **sICAM-1 is a product, marker and mediator of T2DM pathology**  Hyperglycaemia enhances soluble ICAM-1 (sICAM-1) release from activated endothelial cells and thus alters the adhesive properties of the endothelium. This is partly mediated by reduced NO availability (Giugliano et al., 1997, Marfella et al., 2000).  Soluble forms of adhesion molecules provide an index of pathological endothelial activation (Gearing et al., 1992). Consequently, sICAM can be viewed as an independent risk factor or marker for T2DM (Meigs et al., 2006),  Mediates T2DM-associated vascular endothelial dysfunction (Eringa et al., 2013, Kulkarni et al., 2016, Meigs et al., 2006).  Binds monocytes/leukocytes to activated vascular endothelium, antecedent to macrophage and foam cell development (Price and Loscalzo, 1999). |
| **TNF-α** | **TNF-α, a mediator of insulin resistance in insulin target tissue**  TNF*α* stimulates ROS production and reduces NO bioavailability (Kleinbongard et al., 2010, Park et al., 2011).  Favours serine phosphorylation of the insulin receptor and IRS-1 thus disrupting insulin signalling pathway in adipocytes (i.e. reduced adipocyte insulin sensitivity) (Hotamisligil et al., 1994, Hotamisligil et al., 1996, Yuan et al., 2001).  TNF-α directly inhibits hepatic insulin activity resulting in enhanced hepatic glucose production (Ruan and Lodish, 2003).  Directly attenuates glycogen synthesis and glucose uptake by skeletal muscle (Ruan and Lodish, 2003).  TNF-α via NF-κB activation down-regulates adipocyte insulin signalling genes such as IRS-1 and GLUT-4, consequently decreasing glucose uptake in adipocytes (Hauer et al., 2005, Ruan et al., 2002b).  **TNF-α enhances dyslipidaemia in T2DM**  TNF-α induces adipocyte lipolysis via increased adipocyte hormone-sensitive lipase and LpL activity thus increasing FFA levels in circulation and indirectly impairing insulin signalling via FFA-mediated hepatic glucose production and reduced muscle glucose uptake (Abdel-Hamid et al., 2013, Ruan and Lodish, 2003, Zechner et al., 1988).  TNF-α inhibits skeletal muscle fatty acid oxidation contributing to elevated plasma FFA levels (Ruan and Lodish, 2003).  TNF-α promotes hepatic lipogenesis and cholesterol production, including LDL synthesis (Ruan et al., 2002a). |

**REFERENCES**

ABDEL-HAMID, N., JUBORI, T. A., FARHAN, A., MAHROUS, M., GOURI, A., AWAD, E. & BREUSS, J. 2013. Underlying pathways for interferon risk to type II diabetes mellitus. *Curr Diabetes Rev,* 9**,** 472-7.

AKAIWA, M., YU, B., UMESHITA-SUYAMA, R., TERADA, N., SUTO, H., KOGA, T., ARIMA, K., MATSUSHITA, S., SAITO, H., OGAWA, H., FURUE, M., HAMASAKI, N., OHSHIMA, K. & IZUHARA, K. 2001. Localization of human interleukin 13 receptor in non-haematopoietic cells. *Cytokine,* 13**,** 75-84.

ALI, M., MALI, V., HADDOX, S., ABDELGHANY, S. M., EL-DEEK, S. E. M., ABULFADL, A., MATROUGUI, K. & BELMADANI, S. 2017. Essential Role of IL-12 in Angiogenesis in Type 2 Diabetes. *Am J Pathol,* 187**,** 2590-2601.

AMIR LEVY, Y., CIARALDI, T. P., MUDALIAR, S. R., PHILLIPS, S. A. & HENRY, R. R. 2015. Excessive secretion of IL-8 by skeletal muscle in type 2 diabetes impairs tube growth: potential role of PI3K and the Tie2 receptor. *Am J Physiol Endocrinol Metab,* 309**,** E22-34.

ANDRE, P., HARTWELL, D., HRACHOVINOVA, I., SAFFARIPOUR, S. & WAGNER, D. D. 2000. Pro-coagulant state resulting from high levels of soluble P-selectin in blood. *Proc Natl Acad Sci U S A,* 97**,** 13835-40.

BAE, J. S., KIM, Y. U., PARK, M. K. & REZAIE, A. R. 2009. Concentration dependent dual effect of thrombin in endothelial cells via Par-1 and Pi3 Kinase. *J Cell Physiol,* 219**,** 744-51.

BARRY, J. C., SHAKIBAKHO, S., DURRER, C., SIMTCHOUK, S., JAWANDA, K. K., CHEUNG, S. T., MUI, A. L. & LITTLE, J. P. 2016. Hyporesponsiveness to the anti-inflammatory action of interleukin-10 in type 2 diabetes. *Sci Rep,* 6**,** 21244.

BENOIST, C. & MATHIS, D. 1997. Cell death mediators in autoimmune diabetes--no shortage of suspects. *Cell,* 89**,** 1-3.

BESTER, J., MATSHAILWE, C. & PRETORIUS, E. 2018. Simultaneous presence of hypercoagulation and increased clot lysis time due to IL-1beta, IL-6 and IL-8. *Cytokine,* 110**,** 237-242.

BESTER, J. & PRETORIUS, E. 2016. Effects of IL-1beta, IL-6 and IL-8 on erythrocytes, platelets and clot viscoelasticity. *Sci Rep,* 6**,** 32188.

BOKAREWA, M. I., MORRISSEY, J. H. & TARKOWSKI, A. 2002. Tissue factor as a proinflammatory agent. *Arthritis Res,* 4**,** 190-5.

BONI-SCHNETZLER, M., EHSES, J. A., FAULENBACH, M. & DONATH, M. Y. 2008. Insulitis in type 2 diabetes. *Diabetes Obes Metab,* 10 Suppl 4**,** 201-4.

BONIG, H., BURDACH, S., GOBEL, U. & NURNBERGER, W. 2001. Growth factors and hemostasis: differential effects of GM-CSF and G-CSF on coagulation activation--laboratory and clinical evidence. *Ann Hematol,* 80**,** 525-30.

BUSCH, G., SEITZ, I., STEPPICH, B., HESS, S., ECKL, R., SCHOMIG, A. & OTT, I. 2005. Coagulation factor Xa stimulates interleukin-8 release in endothelial cells and mononuclear leukocytes: implications in acute myocardial infarction. *Arterioscler Thromb Vasc Biol,* 25**,** 461-6.

CARLSEN, E., FLATMARK, A. & PRYDZ, H. 1988. Cytokine-induced procoagulant activity in monocytes and endothelial cells. Further enhancement by cyclosporine. *Transplantation,* 46**,** 575-80.

CELI, A., PELLEGRINI, G., LORENZET, R., DE BLASI, A., READY, N., FURIE, B. C. & FURIE, B. 1994. P-selectin induces the expression of tissue factor on monocytes. *Proc Natl Acad Sci U S A,* 91**,** 8767-71.

CERIELLO, A., QUAGLIARO, L., PICONI, L., ASSALONI, R., DA ROS, R., MAIER, A., ESPOSITO, K. & GIUGLIANO, D. 2004. Effect of postprandial hypertriglyceridemia and hyperglycemia on circulating adhesion molecules and oxidative stress generation and the possible role of simvastatin treatment. *Diabetes,* 53**,** 701-10.

CHAN, C. S., MING-LUM, A., GOLDS, G. B., LEE, S. J., ANDERSON, R. J. & MUI, A. L. 2012. Interleukin-10 inhibits lipopolysaccharide-induced tumor necrosis factor-alpha translation through a SHIP1-dependent pathway. *J Biol Chem,* 287**,** 38020-7.

CHANG, Y. H., HO, K. T., LU, S. H., HUANG, C. N. & SHIAU, M. Y. 2012. Regulation of glucose/lipid metabolism and insulin sensitivity by interleukin-4. *Int J Obes (Lond),* 36**,** 993-8.

CHEN, T. C., CHIEN, S. J., KUO, H. C., HUANG, W. S., SHEEN, J. M., LIN, T. H., YEN, C. K., SUNG, M. L. & CHEN, C. N. 2011. High glucose-treated macrophages augment E-selectin expression in endothelial cells. *J Biol Chem,* 286**,** 25564-73.

CROVELLO, C. S., FURIE, B. C. & FURIE, B. 1993. Rapid phosphorylation and selective dephosphorylation of P-selectin accompanies platelet activation. *J Biol Chem,* 268**,** 14590-3.

DANDONA, P., ALJADA, A., MOHANTY, P., GHANIM, H., HAMOUDA, W., ASSIAN, E. & AHMAD, S. 2001. Insulin inhibits intranuclear nuclear factor kappaB and stimulates IkappaB in mononuclear cells in obese subjects: evidence for an anti-inflammatory effect? *J Clin Endocrinol Metab,* 86**,** 3257-65.

DEVRIES, M. E., RAN, L. & KELVIN, D. J. 1999. On the edge: the physiological and pathophysiological role of chemokines during inflammatory and immunological responses. *Semin Immunol,* 11**,** 95-104.

DONATH, M. Y., STORLING, J., MAEDLER, K. & MANDRUP-POULSEN, T. 2003. Inflammatory mediators and islet beta-cell failure: a link between type 1 and type 2 diabetes. *J Mol Med (Berl),* 81**,** 455-70.

DRAGOMIR, E., MANDUTEANU, I., CALIN, M., GAN, A. M., STAN, D., KOENEN, R. R., WEBER, C. & SIMIONESCU, M. 2008. High glucose conditions induce upregulation of fractalkine and monocyte chemotactic protein-1 in human smooth muscle cells. *Thromb Haemost,* 100**,** 1155-65.

DUAN, J., CHUNG, H., TROY, E. & KASPER, D. L. 2010. Microbial colonization drives expansion of IL-1 receptor 1-expressing and IL-17-producing gamma/delta T cells. *Cell Host Microbe,* 7**,** 140-50.

ELHADD, T. A., KENNEDY, G., ROBB, R., MCLAREN, M., JUNG, R. T. & BELCH, J. J. 2004. Elevated soluble cell adhesion molecules E-selectin and intercellular cell adhesion molecule-1 in type-2 diabetic patients with and without asymptomatic peripheral arterial disease. *Int Angiol,* 23**,** 128-33.

EMANUELLI, B., PERALDI, P., FILLOUX, C., SAWKA-VERHELLE, D., HILTON, D. & VAN OBBERGHEN, E. 2000. SOCS-3 is an insulin-induced negative regulator of insulin signaling. *J Biol Chem,* 275**,** 15985-91.

ERINGA, E. C., SERNE, E. H., MEIJER, R. I., SCHALKWIJK, C. G., HOUBEN, A. J., STEHOUWER, C. D., SMULDERS, Y. M. & VAN HINSBERGH, V. W. 2013. Endothelial dysfunction in (pre)diabetes: characteristics, causative mechanisms and pathogenic role in type 2 diabetes. *Rev Endocr Metab Disord,* 14**,** 39-48.

ERNOFSSON, M. & SIEGBAHN, A. 1996. Platelet-derived growth factor-BB and monocyte chemotactic protein-1 induce human peripheral blood monocytes to express tissue factor. *Thromb Res,* 83**,** 307-20.

ERNOFSSON, M., TENNO, T. & SIEGBAHN, A. 1996. Inhibition of tissue factor surface expression in human peripheral blood monocytes exposed to cytokines. *Br J Haematol,* 95**,** 249-57.

FEI, G. Z., HUANG, Y. H., SWEDENBORG, J. & FROSTEGARD, J. 2003. Oxidised LDL modulates immune-activation by an IL-12 dependent mechanism. *Atherosclerosis,* 169**,** 77-85.

FRENETTE, P. S., JOHNSON, R. C., HYNES, R. O. & WAGNER, D. D. 1995. Platelets roll on stimulated endothelium in vivo: an interaction mediated by endothelial P-selectin. *Proc Natl Acad Sci U S A,* 92**,** 7450-4.

FRIED, S. K., APPEL, B. & ZECHNER, R. 1993. Interleukin 1 alpha decreases the synthesis and activity of lipoprotein lipase in human adipose tissue. *Horm Metab Res,* 25**,** 129-30.

FROSTEGARD, J., ULFGREN, A. K., NYBERG, P., HEDIN, U., SWEDENBORG, J., ANDERSSON, U. & HANSSON, G. K. 1999. Cytokine expression in advanced human atherosclerotic plaques: dominance of pro-inflammatory (Th1) and macrophage-stimulating cytokines. *Atherosclerosis,* 145**,** 33-43.

GASPERINI, S., MARCHI, M., CALZETTI, F., LAUDANNA, C., VICENTINI, L., OLSEN, H., MURPHY, M., LIAO, F., FARBER, J. & CASSATELLA, M. A. 1999. Gene expression and production of the monokine induced by IFN-gamma (MIG), IFN-inducible T cell alpha chemoattractant (I-TAC), and IFN-gamma-inducible protein-10 (IP-10) chemokines by human neutrophils. *J Immunol,* 162**,** 4928-37.

GEARING, A. J., HEMINGWAY, I., PIGOTT, R., HUGHES, J., REES, A. J. & CASHMAN, S. J. 1992. Soluble forms of vascular adhesion molecules, E-selectin, ICAM-1, and VCAM-1: pathological significance. *Ann N Y Acad Sci,* 667**,** 324-31.

GERHARDT, C. C., ROMERO, I. A., CANCELLO, R., CAMOIN, L. & STROSBERG, A. D. 2001. Chemokines control fat accumulation and leptin secretion by cultured human adipocytes. *Mol Cell Endocrinol,* 175**,** 81-92.

GIUGLIANO, D., MARFELLA, R., COPPOLA, L., VERRAZZO, G., ACAMPORA, R., GIUNTA, R., NAPPO, F., LUCARELLI, C. & D'ONOFRIO, F. 1997. Vascular effects of acute hyperglycemia in humans are reversed by L-arginine. Evidence for reduced availability of nitric oxide during hyperglycemia. *Circulation,* 95**,** 1783-90.

GLIOZZI, M., GREENWELL-WILD, T., JIN, W., MOUTSOPOULOS, N. M., KAPSOGEORGOU, E., MOUTSOPOULOS, H. M. & WAHL, S. M. 2013. A link between interferon and augmented plasmin generation in exocrine gland damage in Sjogren's syndrome. *J Autoimmun,* 40**,** 122-33.

GOKMEN, A., USTUNDAG, Y., OZTOPRAK, N., AYDEMIR, S., TEKIN, I. O., CAKAL, B., KIRAN, S. & ENGIN, H. 2011. Effects of pegylated interferon alpha on fibrinolytic parameters in patients with chronic hepatitis C. *Clin Appl Thromb Hemost,* 17**,** 449-53.

GOKULAKRISHNAN, K., DEEPA, R., MOHAN, V. & GROSS, M. D. 2006. Soluble P-selectin and CD40L levels in subjects with prediabetes, diabetes mellitus, and metabolic syndrome--the Chennai Urban Rural Epidemiology Study. *Metabolism,* 55**,** 237-42.

GREENBERG, A. S., NORDAN, R. P., MCINTOSH, J., CALVO, J. C., SCOW, R. O. & JABLONS, D. 1992. Interleukin 6 reduces lipoprotein lipase activity in adipose tissue of mice in vivo and in 3T3-L1 adipocytes: a possible role for interleukin 6 in cancer cachexia. *Cancer Res,* 52**,** 4113-6.

GUPTA, S., PABLO, A. M., JIANG, X., WANG, N., TALL, A. R. & SCHINDLER, C. 1997. IFN-gamma potentiates atherosclerosis in ApoE knock-out mice. *J Clin Invest,* 99**,** 2752-61.

HAMILTON, J. A., WHITTY, G. A., STANTON, H., WOJTA, J., GALLICHIO, M., MCGRATH, K. & IANCHES, G. 1993. Macrophage colony-stimulating factor and granulocyte-macrophage colony-stimulating factor stimulate the synthesis of plasminogen-activator inhibitors by human monocytes. *Blood,* 82**,** 3616-21.

HART, P. H., VITTI, G. F., BURGESS, D. R., WHITTY, G. A., ROYSTON, K. & HAMILTON, J. A. 1991. Activation of human monocytes by granulocyte-macrophage colony-stimulating factor: increased urokinase-type plasminogen activator activity. *Blood,* 77**,** 841-8.

HARTMAN, M. E., O'CONNOR, J. C., GODBOUT, J. P., MINOR, K. D., MAZZOCCO, V. R. & FREUND, G. G. 2004. Insulin receptor substrate-2-dependent interleukin-4 signaling in macrophages is impaired in two models of type 2 diabetes mellitus. *J Biol Chem,* 279**,** 28045-50.

HAUER, A. D., UYTTENHOVE, C., DE VOS, P., STROOBANT, V., RENAULD, J. C., VAN BERKEL, T. J., VAN SNICK, J. & KUIPER, J. 2005. Blockade of interleukin-12 function by protein vaccination attenuates atherosclerosis. *Circulation,* 112**,** 1054-62.

HERBERT, J. M., SAVI, P., LAPLACE, M. C. & LALE, A. 1992. IL-4 inhibits LPS-, IL-1 beta- and TNF alpha-induced expression of tissue factor in endothelial cells and monocytes. *FEBS Lett,* 310**,** 31-3.

HERBERT, J. M., SAVI, P., LAPLACE, M. C., LALE, A., DOL, F., DUMAS, A., LABIT, C. & MINTY, A. 1993. IL-4 and IL-13 exhibit comparable abilities to reduce pyrogen-induced expression of procoagulant activity in endothelial cells and monocytes. *FEBS Lett,* 328**,** 268-70.

HONG, E. G., KO, H. J., CHO, Y. R., KIM, H. J., MA, Z., YU, T. Y., FRIEDLINE, R. H., KURT-JONES, E., FINBERG, R., FISCHER, M. A., GRANGER, E. L., NORBURY, C. C., HAUSCHKA, S. D., PHILBRICK, W. M., LEE, C. G., ELIAS, J. A. & KIM, J. K. 2009. Interleukin-10 prevents diet-induced insulin resistance by attenuating macrophage and cytokine response in skeletal muscle. *Diabetes,* 58**,** 2525-35.

HOT, A., LENIEF, V. & MIOSSEC, P. 2012. Combination of IL-17 and TNFalpha induces a pro-inflammatory, pro-coagulant and pro-thrombotic phenotype in human endothelial cells. *Ann Rheum Dis,* 71**,** 768-76.

HOTAMISLIGIL, G. S., BUDAVARI, A., MURRAY, D. & SPIEGELMAN, B. M. 1994. Reduced tyrosine kinase activity of the insulin receptor in obesity-diabetes. Central role of tumor necrosis factor-alpha. *J Clin Invest,* 94**,** 1543-9.

HOTAMISLIGIL, G. S., PERALDI, P., BUDAVARI, A., ELLIS, R., WHITE, M. F. & SPIEGELMAN, B. M. 1996. IRS-1-mediated inhibition of insulin receptor tyrosine kinase activity in TNF-alpha- and obesity-induced insulin resistance. *Science,* 271**,** 665-8.

HOVI, T., SAKSELA, O. & VAHERI, A. 1981. Increased secretion of plasminogen activator by human macrophages after exposure to leukocyte interferon. *FEBS Lett,* 129**,** 233-6.

INOUE, H., OGAWA, W., ASAKAWA, A., OKAMOTO, Y., NISHIZAWA, A., MATSUMOTO, M., TESHIGAWARA, K., MATSUKI, Y., WATANABE, E., HIRAMATSU, R., NOTOHARA, K., KATAYOSE, K., OKAMURA, H., KAHN, C. R., NODA, T., TAKEDA, K., AKIRA, S., INUI, A. & KASUGA, M. 2006. Role of hepatic STAT3 in brain-insulin action on hepatic glucose production. *Cell Metab,* 3**,** 267-75.

JAGER, J., GREMEAUX, T., CORMONT, M., LE MARCHAND-BRUSTEL, Y. & TANTI, J. F. 2007. Interleukin-1beta-induced insulin resistance in adipocytes through down-regulation of insulin receptor substrate-1 expression. *Endocrinology,* 148**,** 241-51.

JANSEN, P. M., BOERMEESTER, M. A., FISCHER, E., DE JONG, I. W., VAN DER POLL, T., MOLDAWER, L. L., HACK, C. E. & LOWRY, S. F. 1995. Contribution of interleukin-1 to activation of coagulation and fibrinolysis, neutrophil degranulation, and the release of secretory-type phospholipase A2 in sepsis: studies in nonhuman primates after interleukin-1 alpha administration and during lethal bacteremia. *Blood,* 86**,** 1027-34.

JIA, H., THELWELL, C., DILGER, P., BIRD, C., DANIELS, S. & WADHWA, M. 2018. Endothelial cell functions impaired by interferon in vitro: Insights into the molecular mechanism of thrombotic microangiopathy associated with interferon therapy. *Thromb Res,* 163**,** 105-116.

JIANG, L. Q., FRANCK, N., EGAN, B., SJOGREN, R. J., KATAYAMA, M., DUQUE-GUIMARAES, D., ARNER, P., ZIERATH, J. R. & KROOK, A. 2013. Autocrine role of interleukin-13 on skeletal muscle glucose metabolism in type 2 diabetic patients involves microRNA let-7. *Am J Physiol Endocrinol Metab,* 305**,** E1359-66.

JONES, C. M., VARESIO, L., HERBERMAN, R. B. & PESTKA, S. 1982. Interferon activates macrophages to produce plasminogen activator. *J Interferon Res,* 2**,** 377-86.

JOSHI-BARVE, S., BARVE, S. S., BUTT, W., KLEIN, J. & MCCLAIN, C. J. 2003. Inhibition of proteasome function leads to NF-kappaB-independent IL-8 expression in human hepatocytes. *Hepatology,* 38**,** 1178-87.

KAMIMURA, M., VIEDT, C., DALPKE, A., ROSENFELD, M. E., MACKMAN, N., COHEN, D. M., BLESSING, E., PREUSCH, M., WEBER, C. M., KREUZER, J., KATUS, H. A. & BEA, F. 2005. Interleukin-10 suppresses tissue factor expression in lipopolysaccharide-stimulated macrophages via inhibition of Egr-1 and a serum response element/MEK-ERK1/2 pathway. *Circ Res,* 97**,** 305-13.

KAMINSKI, A., WELTERS, H. J., KAMINSKI, E. R. & MORGAN, N. G. 2009. Human and rodent pancreatic beta-cells express IL-4 receptors and IL-4 protects against beta-cell apoptosis by activation of the PI3K and JAK/STAT pathways. *Biosci Rep,* 30**,** 169-75.

KANG, K., REILLY, S. M., KARABACAK, V., GANGL, M. R., FITZGERALD, K., HATANO, B. & LEE, C. H. 2008. Adipocyte-derived Th2 cytokines and myeloid PPARdelta regulate macrophage polarization and insulin sensitivity. *Cell Metab,* 7**,** 485-95.

KAPLANSKI, G., FABRIGOULE, M., BOULAY, V., DINARELLO, C. A., BONGRAND, P., KAPLANSKI, S. & FARNARIER, C. 1997. Thrombin induces endothelial type II activation in vitro: IL-1 and TNF-alpha-independent IL-8 secretion and E-selectin expression. *J Immunol,* 158**,** 5435-41.

KAPLANSKI, G., MARIN, V., FABRIGOULE, M., BOULAY, V., BENOLIEL, A. M., BONGRAND, P., KAPLANSKI, S. & FARNARIER, C. 1998. Thrombin-activated human endothelial cells support monocyte adhesion in vitro following expression of intercellular adhesion molecule-1 (ICAM-1; CD54) and vascular cell adhesion molecule-1 (VCAM-1; CD106). *Blood,* 92**,** 1259-67.

KAPPELMAYER, J. & NAGY, B., JR. 2017. The Interaction of Selectins and PSGL-1 as a Key Component in Thrombus Formation and Cancer Progression. *Biomed Res Int,* 2017**,** 6138145.

KIELY, A., MCCLENAGHAN, N. H., FLATT, P. R. & NEWSHOLME, P. 2007. Pro-inflammatory cytokines increase glucose, alanine and triacylglycerol utilization but inhibit insulin secretion in a clonal pancreatic beta-cell line. *J Endocrinol,* 195**,** 113-23.

KIM, D. H., SANDOVAL, D., REED, J. A., MATTER, E. K., TOLOD, E. G., WOODS, S. C. & SEELEY, R. J. 2008. The role of GM-CSF in adipose tissue inflammation. *Am J Physiol Endocrinol Metab,* 295**,** E1038-46.

KIM, T. H., CHOI, S. E., HA, E. S., JUNG, J. G., HAN, S. J., KIM, H. J., KIM, D. J., KANG, Y. & LEE, K. W. 2013. IL-6 induction of TLR-4 gene expression via STAT3 has an effect on insulin resistance in human skeletal muscle. *Acta Diabetol,* 50**,** 189-200.

KISTORP, C., CHONG, A. Y., GUSTAFSSON, F., GALATIUS, S., RAYMOND, I., FABER, J., LIP, G. Y. & HILDEBRANDT, P. 2008. Biomarkers of endothelial dysfunction are elevated and related to prognosis in chronic heart failure patients with diabetes but not in those without diabetes. *Eur J Heart Fail,* 10**,** 380-7.

KLEINBONGARD, P., HEUSCH, G. & SCHULZ, R. 2010. TNFalpha in atherosclerosis, myocardial ischemia/reperfusion and heart failure. *Pharmacol Ther,* 127**,** 295-314.

KLOVER, P. J., ZIMMERS, T. A., KONIARIS, L. G. & MOONEY, R. A. 2003. Chronic exposure to interleukin-6 causes hepatic insulin resistance in mice. *Diabetes,* 52**,** 2784-9.

KOBASHI, C., ASAMIZU, S., ISHIKI, M., IWATA, M., USUI, I., YAMAZAKI, K., TOBE, K., KOBAYASHI, M. & URAKAZE, M. 2009. Inhibitory effect of IL-8 on insulin action in human adipocytes via MAP kinase pathway. *J Inflamm (Lond),* 6**,** 25.

KOUREMBANAS, S., MCQUILLAN, L. P., LEUNG, G. K. & FALLER, D. V. 1993. Nitric oxide regulates the expression of vasoconstrictors and growth factors by vascular endothelium under both normoxia and hypoxia. *J Clin Invest,* 92**,** 99-104.

KRISTIANSEN, O. P. & MANDRUP-POULSEN, T. 2005. Interleukin-6 and diabetes: the good, the bad, or the indifferent? *Diabetes,* 54 Suppl 2**,** S114-24.

KULKARNI, H., MAMTANI, M., PERALTA, J., ALMEIDA, M., DYER, T. D., GORING, H. H., JOHNSON, M. P., DUGGIRALA, R., MAHANEY, M. C., OLVERA, R. L., ALMASY, L., GLAHN, D. C., WILLIAMS-BLANGERO, S., CURRAN, J. E. & BLANGERO, J. 2016. Soluble Forms of Intercellular and Vascular Cell Adhesion Molecules Independently Predict Progression to Type 2 Diabetes in Mexican American Families. *PLoS One,* 11**,** e0151177.

LEE, S. H. & EPSTEIN, L. B. 1980. Reversible inhibition by interferon of the maturation of human peripheral blood monocytes to macrophages. *Cell Immunol,* 50**,** 177-90.

LINDMARK, E., TENNO, T., CHEN, J. & SIEGBAHN, A. 1998. IL-10 inhibits LPS-induced human monocyte tissue factor expression in whole blood. *Br J Haematol,* 102**,** 597-604.

LUMENG, C. N., BODZIN, J. L. & SALTIEL, A. R. 2007. Obesity induces a phenotypic switch in adipose tissue macrophage polarization. *J Clin Invest,* 117**,** 175-84.

MAEDLER, K., SCHUMANN, D. M., SAUTER, N., ELLINGSGAARD, H., BOSCO, D., BAERTSCHIGER, R., IWAKURA, Y., OBERHOLZER, J., WOLLHEIM, C. B., GAUTHIER, B. R. & DONATH, M. Y. 2006. Low concentration of interleukin-1beta induces FLICE-inhibitory protein-mediated beta-cell proliferation in human pancreatic islets. *Diabetes,* 55**,** 2713-22.

MAEDLER, K., SERGEEV, P., RIS, F., OBERHOLZER, J., JOLLER-JEMELKA, H. I., SPINAS, G. A., KAISER, N., HALBAN, P. A. & DONATH, M. Y. 2002. Glucose-induced beta cell production of IL-1beta contributes to glucotoxicity in human pancreatic islets. *J Clin Invest,* 110**,** 851-60.

MANDRUP-POULSEN, T., ZUMSTEG, U., REIMERS, J., POCIOT, F., MORCH, L., HELQVIST, S., DINARELLO, C. A. & NERUP, J. 1993. Involvement of interleukin 1 and interleukin 1 antagonist in pancreatic beta-cell destruction in insulin-dependent diabetes mellitus. *Cytokine,* 5**,** 185-91.

MANETTI, R., PARRONCHI, P., GIUDIZI, M. G., PICCINNI, M. P., MAGGI, E., TRINCHIERI, G. & ROMAGNANI, S. 1993. Natural killer cell stimulatory factor (interleukin 12 [IL-12]) induces T helper type 1 (Th1)-specific immune responses and inhibits the development of IL-4-producing Th cells. *J Exp Med,* 177**,** 1199-204.

MANFREDI, A. A., BALDINI, M., CAMERA, M., BALDISSERA, E., BRAMBILLA, M., PERETTI, G., MASERI, A., ROVERE-QUERINI, P., TREMOLI, E., SABBADINI, M. G. & MAUGERI, N. 2016. Anti-TNFalpha agents curb platelet activation in patients with rheumatoid arthritis. *Ann Rheum Dis,* 75**,** 1511-20.

MARFELLA, R., ESPOSITO, K., GIUNTA, R., COPPOLA, G., DE ANGELIS, L., FARZATI, B., PAOLISSO, G. & GIUGLIANO, D. 2000. Circulating adhesion molecules in humans: role of hyperglycemia and hyperinsulinemia. *Circulation,* 101**,** 2247-51.

MARTINEZ-REYES, C. P., GOMEZ-ARAUZ, A. Y., TORRES-CASTRO, I., MANJARREZ-REYNA, A. N., PALOMERA, L. F., OLIVOS-GARCIA, A., MENDOZA-TENORIO, E., SANCHEZ-MEDINA, G. A., ISLAS-ANDRADE, S., MELENDEZ-MIER, G. & ESCOBEDO, G. 2018. Serum Levels of Interleukin-13 Increase in Subjects with Insulin Resistance but Do Not Correlate with Markers of Low-Grade Systemic Inflammation. 2018**,** 7209872.

MASSBERG, S., GRAHL, L., VON BRUEHL, M. L., MANUKYAN, D., PFEILER, S., GOOSMANN, C., BRINKMANN, V., LORENZ, M., BIDZHEKOV, K., KHANDAGALE, A. B., KONRAD, I., KENNERKNECHT, E., REGES, K., HOLDENRIEDER, S., BRAUN, S., REINHARDT, C., SPANNAGL, M., PREISSNER, K. T. & ENGELMANN, B. 2010. Reciprocal coupling of coagulation and innate immunity via neutrophil serine proteases. *Nat Med,* 16**,** 887-96.

MASSIGNON, D., LEPAPE, A., BIENVENU, J., BARBIER, Y., BOILEAU, C. & COEUR, P. 1994. Coagulation/fibrinolysis balance in septic shock related to cytokines and clinical state. *Haemostasis,* 24**,** 36-48.

MCGILLICUDDY, F. C., CHIQUOINE, E. H., HINKLE, C. C., KIM, R. J., SHAH, R., ROCHE, H. M., SMYTH, E. M. & REILLY, M. P. 2009. Interferon gamma attenuates insulin signaling, lipid storage, and differentiation in human adipocytes via activation of the JAK/STAT pathway. *J Biol Chem,* 284**,** 31936-44.

MEIGS, J. B., O'DONNELL C, J., TOFLER, G. H., BENJAMIN, E. J., FOX, C. S., LIPINSKA, I., NATHAN, D. M., SULLIVAN, L. M., D'AGOSTINO, R. B. & WILSON, P. W. 2006. Hemostatic markers of endothelial dysfunction and risk of incident type 2 diabetes: the Framingham Offspring Study. *Diabetes,* 55**,** 530-7.

MENG, F., WANG, K., AOYAMA, T., GRIVENNIKOV, S. I., PAIK, Y., SCHOLTEN, D., CONG, M., IWAISAKO, K., LIU, X., ZHANG, M., OSTERREICHER, C. H., STICKEL, F., LEY, K., BRENNER, D. A. & KISSELEVA, T. 2012. Interleukin-17 signaling in inflammatory, Kupffer cells, and hepatic stellate cells exacerbates liver fibrosis in mice. *Gastroenterology,* 143**,** 765-776.e3.

MESRI, M. & ALTIERI, D. C. 1999. Leukocyte microparticles stimulate endothelial cell cytokine release and tissue factor induction in a JNK1 signaling pathway. *J Biol Chem,* 274**,** 23111-8.

METZGER, S., NUSAIR, S., PLANER, D., BARASH, V., PAPPO, O., SHILYANSKY, J. & CHAJEK-SHAUL, T. 2004. Inhibition of hepatic gluconeogenesis and enhanced glucose uptake contribute to the development of hypoglycemia in mice bearing interleukin-1beta- secreting tumor. *Endocrinology,* 145**,** 5150-6.

MICHELSON, A. D., BARNARD, M. R., HECHTMAN, H. B., MACGREGOR, H., CONNOLLY, R. J., LOSCALZO, J. & VALERI, C. R. 1996. In vivo tracking of platelets: circulating degranulated platelets rapidly lose surface P-selectin but continue to circulate and function. *Proc Natl Acad Sci U S A,* 93**,** 11877-82.

MILJKOVIC, D., CVETKOVIC, I., MOMCILOVIC, M., MAKSIMOVIC-IVANIC, D., STOSIC-GRUJICIC, S. & TRAJKOVIC, V. 2005. Interleukin-17 stimulates inducible nitric oxide synthase-dependent toxicity in mouse beta cells. *Cell Mol Life Sci,* 62**,** 2658-68.

MOORE, K. W., DE WAAL MALEFYT, R., COFFMAN, R. L. & O'GARRA, A. 2001. Interleukin-10 and the interleukin-10 receptor. *Annu Rev Immunol,* 19**,** 683-765.

MULLARKY, I. K., SZABA, F. M., BERGGREN, K. N., KUMMER, L. W., WILHELM, L. B., PARENT, M. A., JOHNSON, L. L. & SMILEY, S. T. 2006. Tumor necrosis factor alpha and gamma interferon, but not hemorrhage or pathogen burden, dictate levels of protective fibrin deposition during infection. *Infect Immun,* 74**,** 1181-8.

MURRAY, P. J. 2006. STAT3-mediated anti-inflammatory signalling. *Biochem Soc Trans,* 34**,** 1028-31.

NALDINI, A., AARDEN, L., PUCCI, A., BERNINI, C. & CARRARO, F. 2003. Inhibition of interleukin-12 expression by alpha-thrombin in human peripheral blood mononuclear cells: a potential mechanism for modulating Th1/Th2 responses. *Br J Pharmacol,* 140**,** 980-6.

NAWROTH, P. P. & STERN, D. M. 1986. Implication of thrombin formation on the endothelial cell surface. *Semin Thromb Hemost,* 12**,** 197-9.

O'BRIEN, A. D., STANDIFORD, T. J., CHRISTENSEN, P. J., WILCOXEN, S. E. & PAINE, R., 3RD 1998. Chemotaxis of alveolar macrophages in response to signals derived from alveolar epithelial cells. *J Lab Clin Med,* 131**,** 417-24.

OH, Y. S., LEE, Y. J., PARK, E. Y. & JUN, H. S. 2011. Interleukin-6 treatment induces beta-cell apoptosis via STAT-3-mediated nitric oxide production. *Diabetes Metab Res Rev,* 27**,** 813-9.

OLEFSKY, J. M. & GLASS, C. K. 2010. Macrophages, inflammation, and insulin resistance. *Annu Rev Physiol,* 72**,** 219-46.

OSBORN, O., BROWNELL, S. E., SANCHEZ-ALAVEZ, M., SALOMON, D., GRAM, H. & BARTFAI, T. 2008a. Treatment with an Interleukin 1 beta antibody improves glycemic control in diet-induced obesity. *Cytokine,* 44**,** 141-8.

OSBORN, O., GRAM, H., ZORRILLA, E. P., CONTI, B. & BARTFAI, T. 2008b. Insights into the roles of the inflammatory mediators IL-1, IL-18 and PGE2 in obesity and insulin resistance. *Swiss Med Wkly,* 138**,** 665-73.

OSNES, L. T., WESTVIK, A. B., JOO, G. B., OKKENHAUG, C. & KIERULF, P. 1996. Inhibition of IL-1 induced tissue factor (TF) synthesis and procoagulant activity (PCA) in purified human monocytes by IL-4, IL-10 and IL-13. *Cytokine,* 8**,** 822-7.

PAGE, M. J., BESTER, J. & PRETORIUS, E. 2018. The inflammatory effects of TNF-alpha and complement component 3 on coagulation. 8**,** 1812.

PALABRICA, T., LOBB, R., FURIE, B. C., ARONOVITZ, M., BENJAMIN, C., HSU, Y. M., SAJER, S. A. & FURIE, B. 1992. Leukocyte accumulation promoting fibrin deposition is mediated in vivo by P-selectin on adherent platelets. *Nature,* 359**,** 848-51.

PANDEY, M., LOSKUTOFF, D. J. & SAMAD, F. 2005. Molecular mechanisms of tumor necrosis factor-alpha-mediated plasminogen activator inhibitor-1 expression in adipocytes. *Faseb j,* 19**,** 1317-9.

PARK, C., KIM, J. R., SHIM, J. K., KANG, B. S., PARK, Y. G., NAM, K. S., LEE, Y. C. & KIM, C. H. 1999. Inhibitory effects of streptozotocin, tumor necrosis factor-alpha, and interleukin-1beta on glucokinase activity in pancreatic islets and gene expression of GLUT2 and glucokinase. *Arch Biochem Biophys,* 362**,** 217-24.

PARK, Y., YANG, J., ZHANG, H., CHEN, X. & ZHANG, C. 2011. Effect of PAR2 in regulating TNF-alpha and NAD(P)H oxidase in coronary arterioles in type 2 diabetic mice. *Basic Res Cardiol,* 106**,** 111-23.

PETERSEN, E. W., CAREY, A. L., SACCHETTI, M., STEINBERG, G. R., MACAULAY, S. L., FEBBRAIO, M. A. & PEDERSEN, B. K. 2005. Acute IL-6 treatment increases fatty acid turnover in elderly humans in vivo and in tissue culture in vitro. *Am J Physiol Endocrinol Metab,* 288**,** E155-62.

PIEMONTI, L., CALORI, G., LATTUADA, G., MERCALLI, A., RAGOGNA, F., GARANCINI, M. P., RUOTOLO, G., LUZI, L. & PERSEGHIN, G. 2009. Association between plasma monocyte chemoattractant protein-1 concentration and cardiovascular disease mortality in middle-aged diabetic and nondiabetic individuals. *Diabetes Care,* 32**,** 2105-10.

PIEMONTI, L., LEONE, B. E., NANO, R., SACCANI, A., MONTI, P., MAFFI, P., BIANCHI, G., SICA, A., PERI, G., MELZI, R., ALDRIGHETTI, L., SECCHI, A., DI CARLO, V., ALLAVENA, P. & BERTUZZI, F. 2002. Human pancreatic islets produce and secrete MCP-1/CCL2: relevance in human islet transplantation. *Diabetes,* 51**,** 55-65.

POITEVIN, S., COCHERY-NOUVELLON, E., DUPONT, A. & NGUYEN, P. 2007. Monocyte IL-10 produced in response to lipopolysaccharide modulates thrombin generation by inhibiting tissue factor expression and release of active tissue factor-bound microparticles. *Thromb Haemost,* 97**,** 598-607.

PRICE, D. T. & LOSCALZO, J. 1999. Cellular adhesion molecules and atherogenesis. *Am J Med,* 107**,** 85-97.

RABINOVITCH, A. & SUAREZ-PINZON, W. L. 1998. Cytokines and their roles in pancreatic islet beta-cell destruction and insulin-dependent diabetes mellitus. *Biochem Pharmacol,* 55**,** 1139-49.

RAHMAN, A., ANWAR, K. N., TRUE, A. L. & MALIK, A. B. 1999. Thrombin-induced p65 homodimer binding to downstream NF-kappa B site of the promoter mediates endothelial ICAM-1 expression and neutrophil adhesion. *J Immunol,* 162**,** 5466-76.

REED, J. A., CLEGG, D. J., SMITH, K. B., TOLOD-RICHER, E. G., MATTER, E. K., PICARD, L. S. & SEELEY, R. J. 2005. GM-CSF action in the CNS decreases food intake and body weight. *J Clin Invest,* 115**,** 3035-44.

REGNAULT, V., DE MAISTRE, E., CARTEAUX, J. P., GRUEL, Y., NGUYEN, P., TARDY, B. & LECOMPTE, T. 2003. Platelet activation induced by human antibodies to interleukin-8. *Blood,* 101**,** 1419-21.

ROCHA, V. Z., FOLCO, E. J., SUKHOVA, G., SHIMIZU, K., GOTSMAN, I., VERNON, A. H. & LIBBY, P. 2008. Interferon-gamma, a Th1 cytokine, regulates fat inflammation: a role for adaptive immunity in obesity. *Circ Res,* 103**,** 467-76.

ROTTER, V., NAGAEV, I. & SMITH, U. 2003. Interleukin-6 (IL-6) induces insulin resistance in 3T3-L1 adipocytes and is, like IL-8 and tumor necrosis factor-alpha, overexpressed in human fat cells from insulin-resistant subjects. *J Biol Chem,* 278**,** 45777-84.

RUAN, H., HACOHEN, N., GOLUB, T. R., VAN PARIJS, L. & LODISH, H. F. 2002a. Tumor necrosis factor-alpha suppresses adipocyte-specific genes and activates expression of preadipocyte genes in 3T3-L1 adipocytes: nuclear factor-kappaB activation by TNF-alpha is obligatory. *Diabetes,* 51**,** 1319-36.

RUAN, H. & LODISH, H. F. 2003. Insulin resistance in adipose tissue: direct and indirect effects of tumor necrosis factor-alpha. *Cytokine Growth Factor Rev,* 14**,** 447-55.

RUAN, H., MILES, P. D., LADD, C. M., ROSS, K., GOLUB, T. R., OLEFSKY, J. M. & LODISH, H. F. 2002b. Profiling gene transcription in vivo reveals adipose tissue as an immediate target of tumor necrosis factor-alpha: implications for insulin resistance. *Diabetes,* 51**,** 3176-88.

RUTTI, S., HOWALD, C., AROUS, C., DERMITZAKIS, E., HALBAN, P. A. & BOUZAKRI, K. 2016. IL-13 improves beta-cell survival and protects against IL-1beta-induced beta-cell death. *Mol Metab,* 5**,** 122-131.

SAG, D., CARLING, D., STOUT, R. D. & SUTTLES, J. 2008. Adenosine 5'-monophosphate-activated protein kinase promotes macrophage polarization to an anti-inflammatory functional phenotype. *J Immunol,* 181**,** 8633-41.

SARASWATHI, V. & HASTY, A. H. 2006. The role of lipolysis in mediating the proinflammatory effects of very low density lipoproteins in mouse peritoneal macrophages. *J Lipid Res,* 47**,** 1406-15.

SARTIPY, P. & LOSKUTOFF, D. J. 2003. Monocyte chemoattractant protein 1 in obesity and insulin resistance. *Proc Natl Acad Sci U S A,* 100**,** 7265-70.

SATTA, N., TOTI, F., FEUGEAS, O., BOHBOT, A., DACHARY-PRIGENT, J., ESCHWEGE, V., HEDMAN, H. & FREYSSINET, J. M. 1994. Monocyte vesiculation is a possible mechanism for dissemination of membrane-associated procoagulant activities and adhesion molecules after stimulation by lipopolysaccharide. *J Immunol,* 153**,** 3245-55.

SCARPATI, E. M. & SADLER, J. E. 1989. Regulation of endothelial cell coagulant properties. Modulation of tissue factor, plasminogen activator inhibitors, and thrombomodulin by phorbol 12-myristate 13-acetate and tumor necrosis factor. *J Biol Chem,* 264**,** 20705-13.

SCHMID, E., MULLER, T. H., BUDZINSKI, R. M., BINDER, K. & PFIZENMAIER, K. 1995. Signaling by E-selectin and ICAM-1 induces endothelial tissue factor production via autocrine secretion of platelet-activating factor and tumor necrosis factor alpha. *J Interferon Cytokine Res,* 15**,** 819-25.

SCHULTHESS, F. T., PARONI, F., SAUTER, N. S., SHU, L., RIBAUX, P., HAATAJA, L., STRIETER, R. M., OBERHOLZER, J., KING, C. C. & MAEDLER, K. 2009. CXCL10 impairs beta cell function and viability in diabetes through TLR4 signaling. *Cell Metab,* 9**,** 125-39.

SCHWAGER, I. & JUNGI, T. W. 1994. Effect of human recombinant cytokines on the induction of macrophage procoagulant activity. *Blood,* 83**,** 152-60.

SENN, J. J., KLOVER, P. J., NOWAK, I. A. & MOONEY, R. A. 2002. Interleukin-6 induces cellular insulin resistance in hepatocytes. *Diabetes,* 51**,** 3391-9.

SENN, J. J., KLOVER, P. J., NOWAK, I. A., ZIMMERS, T. A., KONIARIS, L. G., FURLANETTO, R. W. & MOONEY, R. A. 2003. Suppressor of cytokine signaling-3 (SOCS-3), a potential mediator of interleukin-6-dependent insulin resistance in hepatocytes. *J Biol Chem,* 278**,** 13740-6.

SERRANO-MARCO, L., BARROSO, E., EL KOCHAIRI, I., PALOMER, X., MICHALIK, L., WAHLI, W. & VAZQUEZ-CARRERA, M. 2012. The peroxisome proliferator-activated receptor (PPAR) beta/delta agonist GW501516 inhibits IL-6-induced signal transducer and activator of transcription 3 (STAT3) activation and insulin resistance in human liver cells. *Diabetologia,* 55**,** 743-51.

SMILEY, S. T., KING, J. A. & HANCOCK, W. W. 2001. Fibrinogen stimulates macrophage chemokine secretion through toll-like receptor 4. *J Immunol,* 167**,** 2887-94.

STANYA, K. J., JACOBI, D., LIU, S., BHARGAVA, P., DAI, L., GANGL, M. R., INOUYE, K., BARLOW, J. L., JI, Y., MIZGERD, J. P., QI, L., SHI, H., MCKENZIE, A. N. & LEE, C. H. 2013. Direct control of hepatic glucose production by interleukin-13 in mice. *J Clin Invest,* 123**,** 261-71.

STEINBERG, H. O., BRECHTEL, G., JOHNSON, A., FINEBERG, N. & BARON, A. D. 1994. Insulin-mediated skeletal muscle vasodilation is nitric oxide dependent. A novel action of insulin to increase nitric oxide release. *J Clin Invest,* 94**,** 1172-9.

STRACZKOWSKI, M., KOWALSKA, I., NIKOLAJUK, A., KRUKOWSKA, A. & GORSKA, M. 2005. Plasma interleukin-10 concentration is positively related to insulin sensitivity in young healthy individuals. *Diabetes Care,* 28**,** 2036-7.

STRANDIN, T., HEPOJOKI, J., LAINE, O., MAKELA, S., KLINGSTROM, J., LUNDKVIST, A., JULKUNEN, I., MUSTONEN, J. & VAHERI, A. 2016. Interferons Induce STAT1-Dependent Expression of Tissue Plasminogen Activator, a Pathogenicity Factor in Puumala Hantavirus Disease. *J Infect Dis,* 213**,** 1632-41.

SUBRAMANIAM, M., FRENETTE, P. S., SAFFARIPOUR, S., JOHNSON, R. C., HYNES, R. O. & WAGNER, D. D. 1996. Defects in hemostasis in P-selectin-deficient mice. *Blood,* 87**,** 1238-42.

SUGIMOTO, R., ENJOJI, M., NAKAMUTA, M., OHTA, S., KOHJIMA, M., FUKUSHIMA, M., KUNIYOSHI, M., ARIMURA, E., MORIZONO, S., KOTOH, K. & NAWATA, H. 2005. Effect of IL-4 and IL-13 on collagen production in cultured LI90 human hepatic stellate cells. *Liver Int,* 25**,** 420-8.

SUHARTI, C., VAN GORP, E. C., SETIATI, T. E., DOLMANS, W. M., DJOKOMOELJANTO, R. J., HACK, C. E., TEN, C. H. & VAN DER MEER, J. W. 2002. The role of cytokines in activation of coagulation and fibrinolysis in dengue shock syndrome. *Thromb Haemost,* 87**,** 42-6.

TAKAHARA, N., KASHIWAGI, A., NISHIO, Y., HARADA, N., KOJIMA, H., MAEGAWA, H., HIDAKA, H. & KIKKAWA, R. 1997. Oxidized lipoproteins found in patients with NIDDM stimulate radical-induced monocyte chemoattractant protein-1 mRNA expression in cultured human endothelial cells. *Diabetologia,* 40**,** 662-70.

TAYLOR-FISHWICK, D. A., WEAVER, J. R., GRZESIK, W., CHAKRABARTI, S., GREEN-MITCHELL, S., IMAI, Y., KUHN, N. & NADLER, J. L. 2013. Production and function of IL-12 in islets and beta cells. *Diabetologia,* 56**,** 126-35.

TOLTL, L. J., BEAUDIN, S. & LIAW, P. C. 2008. Activated protein C up-regulates IL-10 and inhibits tissue factor in blood monocytes. *J Immunol,* 181**,** 2165-73.

TSAO, C. H., SHIAU, M. Y., CHUANG, P. H., CHANG, Y. H. & HWANG, J. 2014. Interleukin-4 regulates lipid metabolism by inhibiting adipogenesis and promoting lipolysis. *J Lipid Res,* 55**,** 385-97.

URBANOVYCH, A., SUSLYK, H. & KOZLOVSKA, K. 2016. Content of sP-selectin and Cytokines in Blood of Patients with Type 2 Diabetes Mellitus and Arterial Hypertension Depending on Diabetes Compensation Condition. . *International Journal of Chemistry,* 8**,** 123.

UYEMURA, K., DEMER, L. L., CASTLE, S. C., JULLIEN, D., BERLINER, J. A., GATELY, M. K., WARRIER, R. R., PHAM, N., FOGELMAN, A. M. & MODLIN, R. L. 1996. Cross-regulatory roles of interleukin (IL)-12 and IL-10 in atherosclerosis. *J Clin Invest,* 97**,** 2130-8.

VAN HALL, G., STEENSBERG, A., SACCHETTI, M., FISCHER, C., KELLER, C., SCHJERLING, P., HISCOCK, N., MOLLER, K., SALTIN, B., FEBBRAIO, M. A. & PEDERSEN, B. K. 2003. Interleukin-6 stimulates lipolysis and fat oxidation in humans. *J Clin Endocrinol Metab,* 88**,** 3005-10.

VASSE, M., PAYSANT, I., SORIA, J., MIRSHAHI, S. S., VANNIER, J. P. & SORIA, C. 1996. Down-regulation of fibrinogen biosynthesis by IL-4, IL-10 and IL-13. *Br J Haematol,* 93**,** 955-61.

VELTROP, M. H., LANGERMANS, J. A., THOMPSON, J. & BANCSI, M. J. 2001. Interleukin-10 regulates the tissue factor activity of monocytes in an in vitro model of bacterial endocarditis. *Infect Immun,* 69**,** 3197-202.

WEAVER, J. R., NADLER, J. L. & TAYLOR-FISHWICK, D. A. 2015. Interleukin-12 (IL-12)/STAT4 Axis Is an Important Element for beta-Cell Dysfunction Induced by Inflammatory Cytokines. *PLoS One,* 10**,** e0142735.

WEN, Y., GU, J., LI, S. L., REDDY, M. A., NATARAJAN, R. & NADLER, J. L. 2006. Elevated glucose and diabetes promote interleukin-12 cytokine gene expression in mouse macrophages. *Endocrinology,* 147**,** 2518-25.

WENG, H. L., LIU, Y., CHEN, J. L., HUANG, T., XU, L. J., GODOY, P., HU, J. H., ZHOU, C., STICKEL, F., MARX, A., BOHLE, R. M., ZIMMER, V., LAMMERT, F., MUELLER, S., GIGOU, M., SAMUEL, D., MERTENS, P. R., SINGER, M. V., SEITZ, H. K. & DOOLEY, S. 2009. The etiology of liver damage imparts cytokines transforming growth factor beta1 or interleukin-13 as driving forces in fibrogenesis. *Hepatology,* 50**,** 230-43.

WOOLLARD, K. J., LUMSDEN, N. G., ANDREWS, K. L., APRICO, A., HARRIS, E., IRVINE, J. C., JEFFERIS, A. M., FANG, L., KANELLAKIS, P., BOBIK, A. & CHIN-DUSTING, J. P. 2014. Raised soluble P-selectin moderately accelerates atherosclerotic plaque progression. *PLoS One,* 9**,** e97422.

YAMAGUCHI, R., YAMAMOTO, T., SAKAMOTO, A., ISHIMARU, Y., NARAHARA, S., SUGIUCHI, H. & YAMAGUCHI, Y. 2016. Substance P enhances tissue factor release from granulocyte-macrophage colony-stimulating factor-dependent macrophages via the p22phox/beta-arrestin 2/Rho A signaling pathway. *Blood Cells Mol Dis,* 57**,** 85-90.

YANG, H., KO, H. J., YANG, J. Y., KIM, J. J., SEO, S. U., PARK, S. G., CHOI, S. S., SEONG, J. K. & KWEON, M. N. 2013. Interleukin-1 promotes coagulation, which is necessary for protective immunity in the lung against Streptococcus pneumoniae infection. *J Infect Dis,* 207**,** 50-60.

YNGEN, M., OSTENSON, C. G., LI, N., HJEMDAHL, P. & WALLEN, N. H. 2001. Acute hyperglycemia increases soluble P-selectin in male patients with mild diabetes mellitus. *Blood Coagul Fibrinolysis,* 12**,** 109-16.

YOUNCE, C. W., WANG, K. & KOLATTUKUDY, P. E. 2010. Hyperglycaemia-induced cardiomyocyte death is mediated via MCP-1 production and induction of a novel zinc-finger protein MCPIP. *Cardiovasc Res,* 87**,** 665-74.

YUAN, M., KONSTANTOPOULOS, N., LEE, J., HANSEN, L., LI, Z. W., KARIN, M. & SHOELSON, S. E. 2001. Reversal of obesity- and diet-induced insulin resistance with salicylates or targeted disruption of Ikkbeta. *Science,* 293**,** 1673-7.

ZECHNER, R., NEWMAN, T. C., SHERRY, B., CERAMI, A. & BRESLOW, J. L. 1988. Recombinant human cachectin/tumor necrosis factor but not interleukin-1 alpha downregulates lipoprotein lipase gene expression at the transcriptional level in mouse 3T3-L1 adipocytes. *Mol Cell Biol,* 8**,** 2394-401.

ZEIGER, F., STEPHAN, S., HOHEISEL, G., PFEIFFER, D., RUEHLMANN, C. & KOKSCH, M. 2000. P-Selectin expression, platelet aggregates, and platelet-derived microparticle formation are increased in peripheral arterial disease. *Blood Coagul Fibrinolysis,* 11**,** 723-8.

ZHANG, H., POTTER, B. J., CAO, J. M. & ZHANG, C. 2011. Interferon-gamma induced adipose tissue inflammation is linked to endothelial dysfunction in type 2 diabetic mice. *Basic Res Cardiol,* 106**,** 1135-45.

ZHANG, H. & ZHANG, C. 2009. Regulation of Microvascular Function by Adipose Tissue in Obesity and Type 2 Diabetes: Evidence of an Adipose-Vascular Loop. *Am J Biomed Sci,* 1**,** 133.

ZHANG, X., NIESSNER, A., NAKAJIMA, T., MA-KRUPA, W., KOPECKY, S. L., FRYE, R. L., GORONZY, J. J. & WEYAND, C. M. 2006. Interleukin 12 induces T-cell recruitment into the atherosclerotic plaque. *Circ Res,* 98**,** 524-31.

ZHANG, Y., ZHOU, B., ZHANG, F., WU, J., HU, Y., LIU, Y. & ZHAI, Q. 2012. Amyloid-beta induces hepatic insulin resistance by activating JAK2/STAT3/SOCS-1 signaling pathway. *Diabetes,* 61**,** 1434-43.

ZHU, Y. P., BROWN, J. R., SAG, D., ZHANG, L. & SUTTLES, J. 2015. Adenosine 5'-monophosphate-activated protein kinase regulates IL-10-mediated anti-inflammatory signaling pathways in macrophages. *J Immunol,* 194**,** 584-94.
